# Development and Psychometric Evaluation of the Peritoneal Dialysis Treatment Adherence Scale: A Multicenter Methodological Study

**DOI:** 10.3390/healthcare14193338

**Published:** 2026-10-07

**Authors:** Zehra Aydın, Tuğba Pehlivan Sarıbudak

**Affiliations:** 1 Department of Nursing, Faculty of Health Sciences, Istanbul Atlas University, 34408 Istanbul, Türkiye; 2 Department of Nursing, Faculty of Health Sciences, Acibadem Mehmet Ali Aydinlar University, 34638 Istanbul, Türkiye

**Keywords:** peritoneal dialysis, treatment adherence, scale development, psychometric properties, knowledge, adherence practices, self-management, nursing assessment

## Abstract

**Highlights:**

**What are the main findings?**

- The Peritoneal Dialysis Treatment Adherence Scale comprises two separately scored dimensions—Knowledge (32 items) and Adherence Practices (25 items)—each covering dialysis treatment, medication use, fluid management, and nutrition.
- Exploratory factor analysis supported four-factor structures for both dimensions. Separate four-factor CFA models showed acceptable fit in the primary analyses; however, ordinal sensitivity analysis indicated estimator-sensitive residual fit, particularly for the Knowledge model, and the integrated eight-factor CFA did not meet all conventional global-fit criteria.

**What are the implications of the main findings?**

- Separate Knowledge and Adherence Practices scores may help healthcare professionals distinguish lower self-reported treatment-related knowledge/awareness from less consistent self-reported adherence practices.
- Domain-specific scores may help identify areas that warrant further individualized patient education and nursing support related to dialysis procedures, medication use, fluid management, and nutrition.

**Abstract:**

**Background/Objectives:** Peritoneal dialysis (PD) requires patients to manage dialysis procedures, medications, fluid intake, and nutrition at home. Existing assessment approaches do not adequately distinguish patients’ treatment-related knowledge and awareness from their self-reported adherence practices across these domains. This study aimed to develop and psychometrically evaluate the Peritoneal Dialysis Treatment Adherence Scale, a patient-reported instrument designed to assess treatment-related knowledge/awareness and self-reported adherence practices in peritoneal dialysis. **Methods:** This multicenter methodological study included 600 adults receiving PD at eight hospitals in Türkiye. An initial 64-item pool was evaluated by 12 experts and pilot-tested with 30 patients. The main sample was randomly divided into two independent subsamples of 300 participants for exploratory factor analysis (EFA) and confirmatory factor analysis (CFA). EFA was conducted using principal axis factoring with direct oblimin rotation, and Horn’s parallel analysis was used to determine factor retention. CFA was conducted in the independent confirmatory subsample. Internal consistency was evaluated using Cronbach’s alpha and McDonald’s omega, and three-week test–retest reliability was assessed in 30 participants using intraclass correlation coefficients. **Results:** The final instrument comprised a 32-item Knowledge Dimension, assessing self-reported treatment knowledge and awareness, and a 25-item Adherence Practices Dimension, assessing the frequency and consistency of self-reported adherence practices. Primary factor loadings ranged from 0.694 to 0.971 for Knowledge and from 0.508 to 0.951 for Adherence Practices, with no items meeting the prespecified criterion for problematic cross-loading. Separate four-factor CFA models demonstrated acceptable within-dimension fit; however, the additional integrated eight-factor CFA including all 57 items did not meet all conventional global-fit criteria, indicating that the overall measurement structure requires further independent validation. Internal consistency in the independent confirmatory subsample was high for both dimensions: Knowledge, α = 0.950 and ω = 0.984; Adherence Practices, α = 0.952 and ω = 0.986. Test–retest reliability was strong for Knowledge (ICC = 0.946) and Adherence Practices (ICC = 0.940). **Conclusions:** The Peritoneal Dialysis Treatment Adherence Scale demonstrated promising but provisional structural evidence, high internal consistency, and strong temporal stability. Its separately scored Knowledge and Adherence Practices dimensions may support domain-specific assessment in PD; however, the overall eight-factor measurement structure requires further independent validation before widespread clinical use.

## 1. Introduction

Peritoneal dialysis (PD) is an established home-based kidney replacement therapy with advantages that include preservation of residual kidney function and better quality of life [1]. Its home-based nature makes sustained self-management particularly important, and contemporary recommendations emphasize goal-directed PD that considers clinical outcomes, treatment burden, patient priorities, and the ability to perform therapy safely at home [2].

The effectiveness of PD depends not only on the prescribed dialysis dose but also on the patient’s ability to carry out a complex self-management regimen. This regimen includes completing dialysis exchanges or automated treatments as prescribed, using medications correctly, monitoring and regulating fluid intake, following nutritional recommendations, maintaining aseptic technique, and responding appropriately to warning signs [2,3]. Because most of these activities occur outside direct professional supervision, difficulties in daily adherence may remain unnoticed and may compromise treatment effectiveness and clinical outcomes [2,3]. Adherence in PD should therefore be understood as a multidimensional and dynamic process rather than as a single behavior [2,3]. Non-adherence occurs across multiple components of PD care. A systematic review identified substantial variation in adherence to dialysis exchanges, medication use, dietary recommendations, and fluid restriction, partly because studies used different definitions and measurement approaches [3]. More recent research in PD has also identified medication non-adherence and associated factors, including age and depressive symptoms [4]. Recent evidence on PD training emphasizes structured and flexible training, assessment of learning outcomes, and retraining when needed [5].

Treatment adherence can be assessed using subjective approaches, such as self-report and questionnaires, and objective indicators, including medication-refill records, biochemical results, dialysis-supply inventories, cycler data, and remote-monitoring records [3,6,7]. Objective indicators may identify missed treatments or abnormal clinical results but do not necessarily explain why they occurred, whereas self-report measures are feasible but vulnerable to recall and social-desirability bias. Renal nurses also report practical barriers to systematic adherence assessment, including time limitations and the underuse of structured patient-reported measures [7]. A clinically useful patient-reported instrument should therefore help identify potential adherence-related difficulties and the specific area requiring further assessment or support.

Existing instruments offer only partial coverage of this need. The End-Stage Renal Disease Adherence Questionnaire assesses several treatment-adherence behaviors but was developed for patients receiving hemodialysis [8]. Other studies in PD have used medication-specific scales, supply inventories, or electronic monitoring approaches [3,4,6]. These methods are valuable for assessing specific behaviors but do not provide an integrated assessment of patients’ treatment-related knowledge and awareness together with their self-reported adherence practices across dialysis treatment, medication use, fluid management, and nutrition. Consequently, a patient may understand what should be done yet not consistently carry out the recommended treatment practices; conversely, a patient may report performing recommended practices while still having gaps in self-reported treatment-related knowledge/awareness. Distinguishing these patterns is important because they require different clinical responses.

The conceptual organization of the proposed instrument was informed by the Health Belief Model and self-efficacy theory. The Health Belief Model proposes that health behavior is influenced by perceived susceptibility, severity, benefits, barriers, and cues to action, whereas self-efficacy reflects confidence in one’s ability to perform a required behavior [9,10]. In PD, these concepts informed a broader distinction between patients’ self-reported knowledge and awareness of treatment requirements and their self-reported implementation of recommended treatment practices. The relevance of self-efficacy to dialysis self-management is also supported by studies in PD and broader dialysis populations [11,12]. The instrument was therefore designed with separate Knowledge and Adherence Practices dimensions. Within each dimension, four clinically relevant adherence domains were specified: dialysis treatment, medication use, fluid management, and nutrition.

This study aimed to develop and psychometrically evaluate the Peritoneal Dialysis Treatment Adherence Scale, a patient-reported instrument assessing treatment-related knowledge/awareness and self-reported adherence practices in adults receiving PD. Specifically, the study examined content validity, item performance, structural validity using exploratory factor analysis (EFA) and confirmatory factor analysis (CFA) in independent samples, internal convergent evidence and within-dimension discriminant validity, internal consistency, and test–retest reliability. It was hypothesized that the Knowledge and Adherence Practices dimensions would each demonstrate a four-factor structure corresponding to dialysis treatment, medication, fluid management, and nutrition, together with acceptable validity and reliability.

## 2. Materials and Methods

### 2.1. Study Design

This multicenter methodological study was conducted to develop and evaluate the psychometric properties of the Peritoneal Dialysis Treatment Adherence Scale. The development and evaluation process comprised construct definition, item generation, expert review, pilot testing, item analysis, EFA, CFA, assessment of internal convergent evidence and within-dimension discriminant validity, internal consistency analysis, and test–retest reliability assessment.

### 2.2. Setting and Participants

The study was conducted in eight peritoneal dialysis centers located in different geographical regions of Türkiye, including four public and four university hospitals. Centers were purposively selected to ensure institutional and geographical diversity and were required to have an established peritoneal dialysis program, sufficient patient volume for recruitment, experienced peritoneal dialysis teams, and willingness to participate in the study. All participating centers had a dedicated peritoneal dialysis outpatient unit, nephrologist oversight, experienced peritoneal dialysis nurses, routine monthly patient follow-up, and facilities for patient education and retraining. These criteria ensured that the participating centers had the core clinical infrastructure and resources necessary for standardized implementation of the study.

The research process was coordinated by the primary study center. The coordinating center developed the study protocol and standardized data-collection materials, briefed the participating peritoneal dialysis nurses and data collectors regarding study procedures, coordinated and monitored data collection across the participating centers, checked completed questionnaires for completeness, and was responsible for data coding, data management, statistical analysis, and overall quality control throughout the study.

All patients receiving peritoneal dialysis at the participating centers who met the eligibility criteria during the data-collection period were invited to participate.

The accessible study population comprised 630 adults receiving peritoneal dialysis. Center-specific recruitment was as follows: Center 1, 80 of 85 eligible patients; Center 2, 74 of 76; Center 3, 80 of 82; Center 4, 66 of 70; Center 5, 84 of 88; Center 6, 90 of 95; Center 7, 61 of 66; and Center 8, 65 of 68. Overall, 600 participants provided informed consent and complete, analyzable data, corresponding to a response rate of 95.2%. Data were collected between 15 May and 30 December 2021 during routine monthly follow-up visits.

Patients were eligible when they were 18 years or older, had been receiving peritoneal dialysis for at least three months, performed their treatment at home, were not hospitalized at the time of data collection, were able to participate in their treatment and self-care activities, and agreed to participate. Patients with cognitive or communication difficulties that prevented them from understanding or responding to the items were excluded.

Literacy was not an exclusion criterion because the instruments were administered through face-to-face interviews. When participants were unable to read independently, trained data collectors read the items and response options verbatim without interpretation or guidance. Only fully completed questionnaires were included in the analyses, and no item-level data imputation was performed.

### 2.3. Sample Size and Sample Allocation

The initial instrument contained 64 items, and the final sample comprised 600 participants. For psychometric evaluation, the sample was randomly divided into two independent subsamples of 300 participants each, allowing exploratory and confirmatory analyses to be conducted in non-overlapping samples. The sample-size decision considered the relatively large number of observed items, the prespecified multidimensional structure, and the planned evaluation of separate four-factor Knowledge and Adherence Practices models alongside an additional integrated eight-factor model. Although no model-specific Monte Carlo simulation or a priori CFA power analysis was performed, the allocation of 300 participants to each subsample was considered a pragmatic approach that enabled independent exploratory and confirmatory analyses. Nevertheless, the adequacy of the confirmatory subsample for the more complex integrated eight-factor model remains uncertain. Sample adequacy was therefore also evaluated empirically by examining factorability, item communalities, factor loadings, and model-fit indices rather than relying solely on a general participant-to-item ratio [13].

Participants were allocated to the two non-overlapping subsamples using the random-number generation function in IBM SPSS Statistics [13]. The first subsample was used for item analysis and EFA, whereas the second was used for CFA and confirmation of internal consistency.

### 2.4. Instrument Development

#### 2.4.1. Conceptual Framework and Item Generation

The Peritoneal Dialysis Treatment Adherence Scale was developed to assess two separately scored dimensions—Knowledge and Adherence Practices—among adults receiving peritoneal dialysis. In this study, treatment adherence refers to the broader clinical process of following the prescribed PD regimen, whereas the two scale dimensions represent distinct patient-reported components of this process: self-reported treatment-related knowledge/awareness and self-reported adherence practices. The term self-management is used only when referring to the broader context of patients’ day-to-day management of PD and is not treated as a separate construct measured by the scale. The Knowledge Dimension reflects self-reported treatment-related knowledge and awareness, including recognition and understanding of PD treatment requirements, recommended practices, and potential consequences of non-adherence. It was designed to capture patient-reported knowledge and awareness rather than objectively tested factual knowledge. The Adherence Practices Dimension was operationalized as a patient-reported construct reflecting the frequency and consistency with which patients reported carrying out recommended PD-related treatment practices. The conceptual framework recognized that adherence-related behavior may be influenced by multiple cognitive and motivational factors, including treatment understanding, beliefs, motivation, and confidence; however, these factors were not operationalized as separate constructs within the Adherence Practices Dimension. Concepts from the Health Belief Model and self-efficacy theory informed the broader conceptual framework of the instrument; however, their individual constructs were not operationalized or evaluated as separate subscales [9,10].

The initial 64-item version administered in the study comprised 39 Knowledge items and 25 Adherence Practices items. The items were generated through a review of the literature on treatment adherence in peritoneal dialysis and other long-term conditions, examination of existing treatment-adherence instruments, consideration of the daily self-management responsibilities associated with peritoneal dialysis, and the clinical experience of the research team [3,4,14,15,16,17]. Within the Knowledge Dimension, the initial items addressed dialysis treatment (15 items), medication use (7 items), fluid management (7 items), and nutrition (10 items). Within the Adherence Practices Dimension, the corresponding domains comprised 13, 3, 6, and 3 items, respectively. Each item was mapped a priori to one of the two intended dimensions and one of the four clinical content domains according to its intended content: items reflecting recognition, understanding, or awareness of treatment requirements were assigned to the Knowledge Dimension, whereas items reflecting the frequency and consistency of self-reported treatment practices were assigned to the Adherence Practices Dimension. This conceptual mapping was specified before factor analysis and was subsequently evaluated empirically through item analysis, EFA, and CFA. The archived development records did not document a separate formal duplicate-item elimination procedure before expert review. The items were originally developed in Turkish and were not translated or adapted from an existing instrument.

#### 2.4.2. Content Validity and Pilot Testing

Content validity was assessed using the Davis technique through structured expert review. Expert judgment and the reporting of item-level and scale-level content validity indices are established components of the content-validation process in scale development [18,19,20]. The expert panel comprised 12 members: 3 nephrologists, 6 nurses with relevant dialysis/nephrology expertise, and 3 academicians with expertise in scale development and measurement. Experts were selected on the basis of their clinical and/or methodological expertise relevant to nephrology, dialysis care, and instrument development. Each item was independently rated on a four-point scale (1 = not appropriate/remove, 2 = not appropriate/major revision required, 3 = appropriate/minor revision required, and 4 = completely appropriate). In accordance with the Davis technique, ratings of 3 or 4 were considered indicative of content adequacy. The item-level Content Validity Index (I-CVI) was calculated as the proportion of the 12 experts assigning either rating to each item. The scale-level Content Validity Index (S-CVI/Ave) was calculated as the mean I-CVI across the 64-item version used in the study. The overall S-CVI/Ave was 0.978, with dimension-specific values of 0.983 for Knowledge and 0.970 for Adherence Practices. Item-level indices ranged from 0.833 to 1.000. Written recommendations regarding wording, clarity, comprehensibility, and content coverage were also reviewed and incorporated where appropriate.

The revised draft instrument was subsequently pilot tested with 30 adults receiving peritoneal dialysis who met the eligibility criteria for the main study [21]. Pilot participants were asked to evaluate the clarity and comprehensibility of the items, the appropriateness of the response options, and the overall ease of completing the instrument. Feedback was reviewed by the research team and considered in relation to recurring difficulties in understanding an item, ambiguity of wording, difficulty selecting a response, or concerns regarding content relevance. No substantial comprehension difficulties or recurring problems requiring item deletion or major modification were identified. Data from the pilot participants were not included in the main psychometric analyses. The pilot testing focused on item clarity, comprehensibility, response options, and ease of completion and did not constitute formal cognitive interviewing. No supplementary objectively scored knowledge items were administered in the original study.

#### 2.4.3. Response Format and Scoring

All items were rated using a five-point Likert-type frequency scale: 1 = Never, 2 = Rarely, 3 = Sometimes, 4 = Often, and 5 = Always.

The scale comprises two separately scored dimensions—Knowledge and Adherence Practices. Each dimension contains four corresponding content domains: dialysis treatment, medication, fluid management, and nutrition. Higher Knowledge scores indicate greater self-reported recognition, understanding, and awareness of PD-related treatment requirements, recommended practices, and potential consequences. These scores should not be interpreted as evidence of objectively tested factual knowledge. Higher Adherence Practices scores indicate greater frequency and consistency of self-reported adherence practices related to recommended PD care.

Following item analysis and exploratory factor analysis, the final instrument comprised 57 items. The Knowledge Dimension contained 32 items, with a theoretical score range of 32–160, and the Adherence Practices Dimension contained 25 items, with a theoretical score range of 25–125. The two dimensions are scored and interpreted separately; no combined total score is calculated across all 57 items.

Negatively worded items in the Knowledge Dimension were reverse-scored as 1→5, 2→4, 3→3, 4→2, and 5→1 before domain and dimension scores were calculated. Domain scores are obtained by summing the items within the corresponding domain, and dimension scores by summing the items within the relevant dimension. For example, if a participant obtains item scores totaling 48 in the 12-item Dialysis Knowledge domain, the domain score is 48. The overall Knowledge Dimension score is then calculated by summing the four Knowledge domain scores. Thus, hypothetical domain scores of 48 for dialysis treatment, 24 for medication use, 20 for fluid management, and 36 for nutrition would yield a total Knowledge score of 128. Negatively worded items are reverse-scored before these sums are calculated. No validated procedure for prorating or imputing missing item responses was established; therefore, domain and dimension scores should be calculated only when all corresponding items are completed. In the absence of validated cutoff values, scores should be interpreted as continuous measures, with higher scores reflecting greater self-reported treatment knowledge/awareness or more consistent self-reported adherence practices, as applicable to the relevant dimension. The complete scale, domain-specific item counts and theoretical score ranges, and scoring guidance are provided in Supplementary Table S5. An overview of the scale-development and psychometric-evaluation process is provided in Supplementary Figure S1.

### 2.5. Data Collection

Data were collected during routine monthly visits to participating peritoneal dialysis centers. Peritoneal dialysis nurses administered the study instruments through individual face-to-face interviews. Before data collection, the nurses received standardized instructions from the investigators regarding participant recruitment, informed consent, questionnaire administration, neutral reading assistance, and completion checks.

Participants were informed about the purpose of the study, the voluntary nature of participation, the confidentiality of their information, and their right to withdraw without any effect on their treatment. Each interview lasted approximately 15 min. Data collectors reviewed the forms immediately after completion to identify unintentionally omitted responses. Participants were invited to complete omitted items but were not directed toward a particular response.

Data were collected using a Participant Information Form and the draft Peritoneal Dialysis Treatment Adherence Scale.

#### 2.5.1. Participant Information Form

The researchers developed the Participant Information Form based on literature examining demographic, clinical, psychosocial, and treatment-related factors associated with adherence in kidney disease and dialysis [22,23,24,25]. It included questions concerning age, sex, marital status, educational level, occupation, living arrangement, perceived economic status, peritoneal dialysis modality, duration of kidney disease, duration of peritoneal dialysis treatment, previous kidney replacement therapy, comorbid conditions, previous education concerning diet and fluid restriction, and self-reported treatment-related behaviors.

#### 2.5.2. Draft Peritoneal Dialysis Treatment Adherence Scale

The draft scale contained 64 items, comprising 39 Knowledge items and 25 Adherence Practices items. Both dimensions included items addressing dialysis treatment, medication use, fluid management, and nutrition.

### 2.6. Statistical Analysis

Data were analyzed using IBM SPSS Statistics version 26 (IBM Corp., Armonk, NY, USA) and IBM SPSS AMOS version 24 (IBM Corp., Armonk, NY, USA). Both software packages were accessed under valid institutional licenses. Statistical tests were two-sided, and *p* < 0.05 was considered statistically significant.

#### 2.6.1. Descriptive Statistics and Item Analysis

Categorical variables were summarized using frequencies and percentages. Continuous variables were summarized using means, standard deviations, minimum and maximum values, skewness, and kurtosis. Distributional normality was assessed using skewness and kurtosis coefficients, with values between −2 and +2 considered indicative of an acceptable approximation to normality [26]. Of the 630 eligible patients, 12 declined participation and 18 were excluded because their questionnaires contained incomplete or missing responses. Only fully completed questionnaires were included in the analyses; consequently, the analytic dataset contained no item-level missing data, and no data imputation was performed.

Floor and ceiling effects were evaluated for the overall Knowledge and Adherence Practices dimension scores by calculating the proportion of participants who achieved the theoretical minimum or maximum possible score. Floor or ceiling effects were considered present when more than 15% of participants achieved the lowest or highest possible score, respectively [27].

Item analysis was conducted separately for the Knowledge and Adherence Practices dimensions in the first subsample of 300 participants. Corrected item–total correlations and Cronbach’s alpha coefficients if an item was deleted were calculated. Items with corrected item–total correlations below 0.30 were considered for removal in conjunction with their conceptual relevance and performance in the factor analysis [13]. The number and original identifiers of the removed items are reported in Section 3.

#### 2.6.2. Exploratory Factor Analysis

Exploratory factor analysis (EFA) was conducted separately for the Knowledge and Adherence Practices dimensions in the first subsample of 300 participants. Sampling adequacy was evaluated using the Kaiser–Meyer–Olkin (KMO) measure, and Bartlett’s test of sphericity was used to assess the factorability of the correlation matrices. Principal axis factoring was used as the common-factor extraction method. Because the adherence domains were theoretically expected to be correlated, direct oblimin rotation was applied [28,29]. The original statistical report identified Pearson product–moment correlations. Accordingly, the EFA is presumed to have used a Pearson correlation matrix rather than a polychoric matrix, although the original EFA syntax was unavailable for independent verification. The number of factors to retain was determined using Horn’s parallel analysis together with examination of factor interpretability and consistency with the prespecified conceptual domains [29]. A primary factor loading of ≥0.40 was considered acceptable. Secondary loadings of ≥0.30 were examined as potential cross-loadings, and an item was considered to show problematic cross-loading when the difference between its primary and secondary loadings was <0.20 [28,29]. Item communalities were also examined as indicators of the proportion of item variance explained by the common factors [28,29]. Item-retention decisions were based on statistical performance together with conceptual relevance and preservation of clinically meaningful domain coverage. The complete pattern matrices, including primary and secondary loadings and communalities, were examined for both dimensions.

#### 2.6.3. Confirmatory Factor Analysis

Confirmatory factor analysis (CFA) was conducted in the independent second subsample of 300 participants. First, separate four-factor CFA models were evaluated for the Knowledge (32 items) and Adherence Practices (25 items) dimensions, each comprising dialysis treatment, medication use, fluid management, and nutrition as correlated latent factors. The separate four-factor CFA models included three within-domain correlated residual pairs in each dimension. In the Knowledge model, these involved original item pairs 11–14 within dialysis treatment, 17–18 within medication use, and 34–38 within nutrition. In the Adherence Practices model, they involved item pairs 5–6 and 11–13 within dialysis treatment and items 5–6 within fluid management. These residual associations were confined to items within the same clinical domain and were substantively interpretable as local dependence between items addressing closely related aspects of the same treatment behavior or requirement. The corresponding residual paths are displayed in Supplementary Figures S2 and S3. Subsequently, an additional integrated eight-factor CFA model including all 57 retained items was evaluated to examine the joint measurement structure of the two dimensions. Each item was specified to load on its prespecified domain, while correlations among all eight latent factors were freely estimated. Knowledge and Adherence Practices remained conceptually distinct and separately scored dimensions; therefore, specification of the integrated CFA did not imply calculation of a combined total score.

The initial integrated model was estimated without correlated residuals or post hoc error covariances. Model fit was evaluated using the chi-square statistic relative to degrees of freedom (χ^2^/df), comparative fit index (CFI), Tucker–Lewis index (TLI), root mean square error of approximation (RMSEA), and standardized root mean square residual (SRMR) [29,30]. The analyses were performed in AMOS 24. Maximum likelihood is the default estimator in AMOS; however, the original estimation setting was not preserved in the available statistical records and could not be verified retrospectively. Model-fit criteria were χ^2^/df below 5, CFI and TLI above 0.90, and RMSEA and SRMR of 0.08 or lower. The five-category Likert-type responses were treated as numerical observed indicators. Because the original CFA treated the five-category Likert-type responses as numerical observed indicators, an additional ordinal sensitivity analysis was subsequently performed, as described below. The potential implications of this approach, particularly given the negative skewness of several Knowledge domains, are acknowledged as a limitation. Standardized factor loadings and latent-factor correlations were examined, and modification indices were reviewed diagnostically for evidence of potentially misspecified cross-dimensional loadings or local item dependence. Model parameters were not freed solely to improve global fit. Detailed standardized factor loadings and latent-factor correlations for the integrated model are provided in Supplementary Table S7.

As a sensitivity analysis addressing the ordinal five-category response format, the CFA models were additionally re-estimated using the polychoric correlation matrix of the independent confirmatory subsample and unweighted least squares (ULS) estimation. The same prespecified factor structures were retained as in the primary CFA analyses. Because the sensitivity analysis used a different treatment of the item responses and estimation approach from the original AMOS analysis, its results were examined primarily to assess the stability of the standardized factor-loading pattern and residual model fit rather than to replace the original CFA findings.

#### 2.6.4. Internal Convergent and Within-Dimension Discriminant Validity

We evaluated internal convergent evidence and within-dimension discriminant validity separately within the Knowledge and Adherence Practices dimensions. CR, AVE, MSV, and ASV were calculated from the separate four-factor CFA models for the Knowledge and Adherence Practices dimensions, respectively, rather than from the integrated eight-factor model. Internal convergent evidence was evaluated using composite reliability (CR) and average variance extracted (AVE). CR values of at least 0.70 and AVE values of at least 0.50 were considered supportive of internal convergent evidence [31,32].

Within-dimension discriminant validity was evaluated using maximum shared variance (MSV), average shared variance (ASV), and inter-factor correlations. Within-dimension discriminant validity was considered supported when MSV and ASV were lower than the corresponding AVE and when the square root of the AVE for each factor exceeded its correlations with the remaining factors within the same dimension [31,32].

#### 2.6.5. Internal Consistency

Internal consistency of the final instrument was evaluated in the independent confirmatory subsample (*n* = 300) using Cronbach’s alpha (α) and McDonald’s omega (ω) coefficients [33,34,35]. Reliability was examined for the overall Knowledge and Adherence Practices dimensions and for each of their four domains. McDonald’s omega for the overall dimensions was estimated from the corresponding four-factor structure, whereas domain-level omega estimates were based on the respective domain models. Ninety-five percent confidence intervals for both α and ω were obtained using participant-level nonparametric bootstrap resampling (1000 resamples). Values of approximately 0.70 or higher were considered indicative of acceptable internal consistency [33,34]. Coefficients exceeding approximately 0.95 were interpreted cautiously because very high reliability may also reflect substantial similarity or redundancy among items. To further examine potential item redundancy, within-domain inter-item correlations were examined in the confirmatory subsample, including the mean and maximum pairwise correlation within each of the eight domains.

#### 2.6.6. Test–Retest Reliability

Temporal stability was assessed in a subsample of 30 participants randomly selected from the main study sample across several participating peritoneal dialysis centers. Participants completed the final 57-item instrument again after a three-week interval. The reported test–retest coefficients correspond to two-way mixed-effects, consistency, average-measures intraclass correlation coefficients [ICC(3, 2)], based on the two administrations, with 95% confidence intervals [36]. Mean scores at both administrations were also summarized. No formal comparison of demographic or clinical characteristics was performed between the test–retest subsample and the full study sample.

#### 2.6.7. Exploratory Group Comparisons

Knowledge and Adherence Practices dimension and domain scores were compared according to participant characteristics using the full sample of 600 participants. Independent-samples t tests were used for variables with two groups, and one-way analysis of variance was used for variables with three or more groups. When an overall analysis of variance result was statistically significant, Tukey’s post hoc test was used to identify differences between groups.

Effect sizes were calculated as Cohen’s d for comparisons involving two groups and partial eta-squared (ηp^2^) for one-way ANOVA comparisons involving three or more groups. Tukey adjustment was applied to post hoc pairwise comparisons following significant ANOVA results; however, no global correction for multiple testing (e.g., Bonferroni correction) was applied across the full set of exploratory demographic and clinical comparisons. Accordingly, these secondary analyses were interpreted as exploratory and hypothesis-generating.

Exact *p*-values were reported where available, and *p* < 0.001 was used instead of *p* = 0.000.

Because measurement invariance was not established across demographic, clinical, or institutional subgroups, these group comparisons were interpreted as exploratory and hypothesis-generating and should not be regarded as evidence of measurement-equivalent between-group differences.

### 2.7. Ethical Considerations

The study was conducted in accordance with the Declaration of Helsinki and was approved by the University Institute of Health Sciences Human Research Ethics Committee on 15 September 2020 (Protocol No. 88). Written informed consent was obtained from all participants before data collection. Participation was voluntary, and declining or withdrawing from the study did not affect the treatment or care received.

### 2.8. Use of Generative Artificial Intelligence

OpenAI ChatGPT (GPT-5.6 Thinking, OpenAI, San Francisco, CA, USA) was used solely for English-language refinement during manuscript preparation. It was not used to generate, modify, analyze, or interpret the study data or the original AMOS model outputs. All generated text was reviewed and revised by the authors, who take full responsibility for the accuracy, originality, and integrity of the manuscript.

## 3. Results

### 3.1. Participant Flow and Characteristics

Of the 630 eligible patients invited to participate, 600 provided complete and analyzable data and were included in the study, corresponding to a response rate of 95.2%. The mean age of the participants was 49.34 ± 13.43 years, with an age range of 23–79 years. Most participants were women (67.0%), married (76.8%), and had completed primary education (48.0%). Approximately two-thirds lived with their spouse and children (64.0%), and 50.7% described their economic status as moderate.

Continuous ambulatory peritoneal dialysis was used by 425 participants (70.8%), whereas 175 participants (29.2%) received automated peritoneal dialysis. The mean duration of kidney disease was 8.62 ± 5.57 years, and the mean duration of peritoneal dialysis treatment was 4.42 ± 3.42 years. More than half of the participants had not previously received another form of kidney replacement therapy (58.8%), whereas 33.2% had previously received hemodialysis and 8.0% had undergone kidney transplantation.

Most participants reported having received information about dietary and fluid restrictions (71.8%). In addition, 70.8% reported following dietary recommendations, 86.7% reported taking their medications regularly, 97.7% reported performing their dialysis treatment regularly, and 87.5% reported routinely monitoring their fluid intake and output. The sociodemographic and clinical characteristics of the participants are presented in Table 1.

### 3.2. Content Validity and Pilot Testing Findings

The initial 64-item instrument was evaluated by 12 experts. The expert evaluation yielded an overall S-CVI/Ave of 0.978, with dimension-specific values of 0.983 for Knowledge and 0.970 for Adherence Practices. Item-level CVI values ranged from 0.833 to 1.000. No items were removed following the expert review.

The draft instrument was subsequently pilot tested with 30 adults receiving peritoneal dialysis. Participants did not report substantial difficulties regarding the clarity, comprehensibility, or response process, and no major revisions were required following pilot testing. Data from the pilot participants were excluded from the main psychometric analyses.

### 3.3. Item Analysis and Item Reduction

Item analysis was conducted separately for the Knowledge and Adherence Practices dimensions in the first subsample of 300 participants. Seven items from the original 39-item knowledge dimensions (4, 5, 12, 16, 23, 26, and 37) were removed because they did not meet the prespecified item-retention criteria.

Following item removal, the corrected item–total correlations of the retained 32 Knowledge items ranged from 0.679 to 0.951. No items were removed from the original 25-item Adherence Practices dimension, for which corrected item–total correlations ranged from 0.667 to 0.962.

The final instrument therefore comprised 57 items: 32 items in the Knowledge dimension and 25 items in the Adherence Practices dimension. The item-reduction process is summarized in Table 2. Detailed item-level results are provided in Supplementary Tables S1 and S2.

### 3.4. Exploratory Factor Analysis (EFA) Findings

Exploratory factor analysis using principal axis factoring with direct oblimin rotation was conducted separately for the Knowledge and Adherence Practices dimensions in the exploratory subsample (*n* = 300). For the Knowledge Dimension, the Kaiser–Meyer–Olkin value was 0.935, and Bartlett’s test of sphericity was statistically significant, χ^2^(496) = 18,011.53, *p* < 0.001, supporting the factorability of the correlation matrix. Horn’s parallel analysis supported retention of four factors, corresponding to dialysis treatment, medication use, fluid management, and nutrition. Primary factor loadings for the 32 retained Knowledge items ranged from 0.694 to 0.971, and communalities ranged from 0.649 to 0.958. Two items had secondary loadings ≥0.30; however, in both cases the difference between the primary and secondary loadings exceeded 0.20, and neither met the predefined criterion for problematic cross-loading. Therefore, no additional Knowledge items were removed.

For the Adherence Practices Dimension, the Kaiser–Meyer–Olkin value was 0.885, and Bartlett’s test of sphericity was statistically significant, χ^2^(300) = 6103.43, *p* < 0.001. Parallel analysis similarly supported a four-factor solution corresponding to dialysis treatment, medication use, fluid management, and nutrition. Primary factor loadings for the 25 Adherence Practices items ranged from 0.508 to 0.951, and communalities ranged from 0.263 to 0.909. No problematic cross-loadings were identified according to the predefined criteria. Although one item had a comparatively low communality, its primary loading exceeded 0.50, it showed no meaningful cross-loading, and it was retained because of its conceptual relevance. Overall, the common-factor analyses supported the prespecified four-domain structure of both dimensions without requiring further item deletion.

The exploratory factor analysis findings are summarized in Table 2, and the complete pattern matrices and communalities are presented in Supplementary Table S6.

### 3.5. Confirmatory Factor Analysis (CFA)

The separate four-factor CFA models demonstrated acceptable within-dimension fit. For Knowledge, χ^2^/df was 2.32, CFI was 0.957, TLI was 0.944, and RMSEA was 0.073. For Adherence Practices, the corresponding values were 2.21, 0.950, 0.913, and 0.067, respectively. The fit indices for both separate models are presented in Table 3 (Panel A). The standardized factor loadings and latent-factor correlations for the separate four-factor Knowledge and Adherence Practices models are presented in Supplementary Figure S2 and Figure S3, respectively.

An integrated eight-factor CFA including all 57 retained items was tested in the independent confirmatory subsample (*n* = 300). The model simultaneously represented the four Knowledge domains and the four corresponding Adherence Practices domains, with correlations freely estimated among the eight latent factors. All items were specified to load only on their prespecified domains.

The initial integrated model, estimated without correlated residuals, yielded χ^2^/df = 4.22, CFI = 0.818, TLI = 0.808, RMSEA = 0.104, and SRMR = 0.067. Although the standardized item loadings were generally strong, the overall CFI, TLI, and RMSEA values did not meet the prespecified criteria for acceptable global model fit. Thus, the integrated analysis did not support the overall eight-factor measurement structure according to conventional global-fit criteria, despite generally strong standardized item loadings.

Modification indices were examined diagnostically; however, extensive post hoc residual covariances were not introduced solely to improve model fit because this could increase the risk of data-driven overfitting. The 57-item structure was therefore retained on the basis of the EFA findings, item performance, conceptual relevance, and preservation of clinically meaningful domain coverage, while the limitations of the integrated CFA fit were acknowledged. Detailed standardized factor loadings and latent-factor correlations are provided in Supplementary Table S7.

In the ordinal sensitivity analysis based on polychoric correlations and ULS estimation, standardized factor loadings remained consistently strong, ranging approximately from 0.75 to 0.99 for the Knowledge model, 0.81 to 0.98 for the Adherence Practices model, and 0.75 to 0.99 for the integrated eight-factor model. However, residual model fit was not fully consistent with the original AMOS results. The ULS-based SRMR values were approximately 0.121 for the Knowledge model, 0.049 for the Adherence Practices model, and 0.080 for the integrated model. Thus, the strong item–factor relationships were broadly stable, whereas residual fit was partly sensitive to the treatment of the ordinal indicators. Accordingly, the CFA findings should be interpreted as provisional structural evidence rather than definitive confirmation of the measurement structure.

### 3.6. Internal Convergent Evidence and Within-Dimension Discriminant Validity

For the knowledge dimension, composite reliability values ranged from 0.953 to 0.980, and average variance extracted values ranged from 0.803 to 0.874. Maximum shared variance values ranged from 0.343 to 0.425, whereas average shared variance values ranged from 0.243 to 0.328. For all knowledge factors, the average variance extracted exceeded the corresponding maximum and average shared variance values.

For the Adherence Practices dimension, composite reliability values ranged from 0.940 to 0.980, and average variance extracted values ranged from 0.779 to 0.901. Maximum shared variance values ranged from 0.078 to 0.272, and average shared variance values ranged from 0.058 to 0.205.

For both dimensions, composite reliability values exceeded 0.70, average variance extracted values exceeded 0.50, and the square root of the average variance extracted for each factor exceeded its correlations with the remaining factors. These findings provided internal convergent evidence and evidence of within-dimension discriminant validity for the Knowledge and Adherence Practices models. Table 4 presents detailed results.

### 3.7. Internal Consistency Findings

Internal consistency of the final instrument was examined in the independent confirmatory subsample (*n* = 300). Cronbach’s alpha and McDonald’s omega coefficients indicated high internal consistency for both dimensions and their respective domains. For the Knowledge Dimension, overall α was 0.950 (95% CI: 0.940–0.956) and ω was 0.984 (95% CI: 0.981–0.987). For the Adherence Practices Dimension, overall α was 0.952 (95% CI: 0.941–0.960) and ω was 0.986 (95% CI: 0.983–0.989). Domain-level coefficients were also high, although several estimates exceeded 0.95 and were therefore interpreted cautiously because very high internal consistency may also indicate overlapping item content. Complete reliability estimates are presented in Table 5. Within-domain inter-item correlations were also examined to assess potential item redundancy. Mean inter-item correlations ranged from approximately 0.78 to 0.92 across the eight domains. The strongest clustering was observed in the Adherence Practices fluid-management domain, where the mean inter-item correlation was approximately 0.92 and the maximum pairwise correlation was approximately 0.97. Several very high pairwise correlations were also observed in the Adherence Practices dialysis-treatment and Knowledge dialysis-treatment domains.

### 3.8. Test–Retest Reliability Findings

Test–retest reliability was evaluated in 30 participants who completed the final instrument twice at a three-week interval.

For the knowledge dimension, the overall intraclass correlation coefficient was 0.946 (95% CI: 0.886–0.974; F = 18.426; *p* < 0.001). Domain-specific intraclass correlation coefficients were 0.857 for dialysis treatment, 0.931 for medication use, 0.909 for fluid management, and 0.962 for nutrition. The mean item score for the knowledge dimension was 4.35 ± 0.16 at the first administration and 4.38 ± 0.20 at the second administration.

For the Adherence Practices dimension, the overall intraclass correlation coefficient was 0.940 (95% CI: 0.875–0.972; F = 16.751; *p* < 0.001). Domain-specific intraclass correlation coefficients were 0.942 for dialysis treatment, 0.845 for medication use, 0.936 for fluid management, and 0.841 for nutrition. The mean item score for the Adherence Practices dimension was 4.21 ± 0.22 at the first administration and 4.19 ± 0.33 at the second administration.

These findings indicated strong temporal stability for the Knowledge and Adherence Practices dimensions and their respective domains. The test–retest reliability results are presented in Table 5.

### 3.9. Dimension and Domain Score Distributions

The mean Knowledge Dimension score was 128.70 ± 31.77 within a theoretical score range of 32–160. Mean domain scores were 50.99 ± 17.71 for dialysis treatment, 22.62 ± 9.21 for medication use, 19.70 ± 6.51 for fluid management, and 35.39 ± 11.83 for nutrition.

The mean Adherence Practices Dimension score was 77.82 ± 27.15 within a theoretical score range of 25–125. Mean domain scores were 38.42 ± 20.47 for dialysis treatment, 11.41 ± 3.83 for medication use, 18.05 ± 8.18 for fluid management, and 9.93 ± 4.57 for nutrition.

Across the dimension and domain scores, skewness values ranged from −1.548 to 0.206 and kurtosis values ranged from −1.651 to 0.947. All values were within the predefined ±2 criterion for an acceptable approximation to normality. Nevertheless, the Knowledge Dimension and its domains showed a more pronounced negative skewness, indicating a greater concentration of scores toward the upper end of their theoretical ranges, whereas Adherence Practices scores showed greater variability across participants. Detailed descriptive statistics are presented in Table 6.

At the dimension level, no participant achieved the theoretical minimum Knowledge score (0.0%), whereas sensitivity assessment of the available raw data indicated that the proportion achieving the theoretical maximum Knowledge score could not exceed 2.7%. For the Adherence Practices Dimension, no participant achieved either the theoretical minimum or maximum score (0/600, 0.0% for both). These findings indicated no substantial floor or ceiling effects for either overall dimension.

### 3.10. Exploratory Comparisons According to Participant Characteristics

Exploratory comparisons according to participant characteristics were conducted using the full sample of 600 participants. Several differences were observed across Knowledge and Adherence Practices dimension and domain scores according to selected demographic and clinical characteristics. Because these analyses were secondary, were not based on prespecified hypotheses, and involved multiple comparisons without a global multiplicity adjustment, the findings should be interpreted as hypothesis-generating and not as evidence of known-groups validity. Detailed comparison results and post hoc findings are provided in Supplementary Tables S3 and S4.

## 4. Discussion

### 4.1. Principal Findings

This multicenter methodological study developed and evaluated the psychometric properties of the Peritoneal Dialysis Treatment Adherence Scale among adults receiving peritoneal dialysis (PD). The final instrument comprises two separately scored dimensions: a 32-item Knowledge dimension and a 25-item Adherence Practices dimension. Each dimension contains four domains representing the major components of PD self-management: dialysis treatment, medication use, fluid management, and nutrition. EFA supported four-factor structures within both dimensions, and the originally specified separate CFA models demonstrated acceptable within-dimension fit; however, the integrated eight-factor model did not meet all conventional global-fit criteria. The findings also provided internal convergent evidence and evidence of within-dimension discriminant validity, high internal consistency, and strong three-week test–retest reliability.

The distinction between the Knowledge and Adherence Practices dimensions is an important feature of the instrument. Adherence in PD is multidimensional and may be affected by different mechanisms. Some patients may report limited self-reported treatment-related knowledge/awareness, whereas others may understand the recommendations but not consistently implement the recommended treatment practices. Evidence from broader kidney populations similarly indicates that medication beliefs, symptom burden, and the practical organization of daily routines may influence adherence [37,38]. Previous PD studies have documented considerable variation in adherence to dialysis procedures, medication use, dietary recommendations, and fluid restrictions and have distinguished between intentional and unintentional non-adherence [3,14]. Recent evidence has also demonstrated variation in medication adherence among patients receiving PD and identified patient- and treatment-related factors associated with adherence [4]. Assessing self-reported treatment knowledge/awareness and self-reported adherence practices separately may therefore provide more clinically informative results than combining all items into a single total score.

### 4.2. Factor Structure and Construct Validity

The four domains identified within both dimensions are consistent with the core responsibilities involved in home PD. Patients are expected to perform dialysis procedures as prescribed, use medications correctly, regulate fluid intake and output, and follow individualized nutritional recommendations [2,3]. The presence of these domains in both the Knowledge and Adherence Practices dimensions is conceptually meaningful because a patient’s understanding of a treatment requirement may differ from the consistency with which the corresponding treatment practice is implemented.

The structural characteristics of the instrument were examined using independent exploratory and confirmatory subsamples. In the exploratory subsample, principal axis factoring with direct oblimin rotation and Horn’s parallel analysis supported four-factor solutions for both the Knowledge and Adherence Practices dimensions. The retained items demonstrated generally strong primary factor loadings, and no items met the prespecified criterion for problematic cross-loading. These findings were consistent with the proposed conceptual organization of dialysis treatment, medication use, fluid management, and nutrition within each dimension [28,29,39]. Use of a common-factor extraction method with oblique rotation was particularly appropriate because the adherence domains were theoretically expected to be related rather than statistically independent.

Because the separate models included a limited number of within-domain correlated residuals whereas the integrated model did not, the comparatively better fit of the separate models should be interpreted with this difference in model specification in mind. An additional integrated eight-factor CFA including all 57 retained items was subsequently conducted to evaluate the overall measurement structure and the relationships among the Knowledge and Adherence Practices domains within a single model. Although standardized item loadings were generally strong, the integrated model did not meet all conventional global-fit criteria. Accordingly, the integrated CFA should not be interpreted as supporting the overall eight-factor measurement structure; rather, the present structural evidence is more consistent with the separate four-factor models for the Knowledge and Adherence Practices dimensions. Thus, the present data provide stronger support for the separate within-dimension structures of Knowledge and Adherence Practices than for a unified eight-factor measurement structure encompassing both dimensions. The integrated model therefore requires further evaluation in an independent population before its generalizability can be established. The use of separate exploratory and confirmatory subsamples nevertheless reduced the likelihood that the identified structure reflected only the characteristics of a single sample and is consistent with recommended scale-development practices [13]. The ordinal sensitivity analysis further indicated that the standardized item–factor relationships remained strong, but model-fit conclusions were not completely invariant to the estimation approach. In particular, residual fit for the Knowledge model was less favorable when the ordinal response structure was accommodated using polychoric correlations and ULS estimation. This estimator sensitivity reinforces the need to regard the present CFA findings as provisional and to replicate the factor structure using ordinal-data estimators in an independent sample.

The instrument differs from existing renal adherence measures in both its target population and conceptual scope. The End-Stage Renal Disease Adherence Questionnaire evaluates adherence to dialysis attendance, medication use, fluid restriction, and dietary recommendations but was developed for patients receiving in-center hemodialysis [8]. The Home Dialysis Care Experience instrument was developed for patients receiving PD or home hemodialysis but measures patients’ experiences of home dialysis care rather than adherence-related knowledge and adherence practices [40]. The use of parallel analysis as an adjunct to factor-retention decisions is also consistent with contemporary factor-analytic methodology [39]. The present instrument may therefore address a more specific clinical need by separately assessing self-reported treatment-related knowledge/awareness and self-reported adherence practices across four PD treatment domains. Nevertheless, its incremental value should be established through direct comparison with existing adherence measures and relevant clinical indicators. Psychometric performance can also be considered in relation to other renal adherence measures. The 13-item Adherence to Healthy Behaviors Scale, developed for patients with chronic kidney disease not requiring dialysis, demonstrated a three-factor structure explaining 47.16% of the variance, a Cronbach’s alpha of 0.70, criterion-related validity of r = 0.51, and test–retest reliability of r = 0.70 [16]. The CKD Knowledge, Attitude, and Practices questionnaire reported Cronbach’s alpha coefficients of 0.963 for knowledge, 0.829 for attitude, and 0.756 for practice [17]. In comparison, the present PD-specific instrument demonstrated high internal consistency and temporal stability, with test–retest ICCs of 0.946 for the Knowledge Dimension and 0.940 for the Adherence Practices Dimension. However, these differences should not be interpreted as evidence of psychometric superiority because the instruments differ substantially in their target populations, constructs, item content, and response formats. Direct comparative validation in patients receiving PD is therefore still required.

The internal convergent evidence reported in this study was based on composite reliability and average variance extracted within the CFA models. Convergent validity against an external measure was not examined. Similarly, criterion-related validity was not evaluated because no established PD-specific adherence measure, objective adherence indicator, or clinical reference standard was included in the original study protocol. Accordingly, the present findings should be interpreted as providing provisional internal structural evidence together with evidence of reliability, rather than as comprehensive external validation of the instrument. Furthermore, discriminant validity between corresponding Knowledge and Adherence Practices domains was not formally established; given the suboptimal fit of the integrated eight-factor model, this aspect of construct validity requires further investigation in an independent sample. Future studies should examine associations with validated adherence measures and objective indicators such as medication-refill data, dialysis records, cycler or remote-monitoring data, biochemical measures, and relevant clinical outcomes.

### 4.3. Internal Consistency and Temporal Stability

Cronbach’s alpha and McDonald’s omega coefficients demonstrated high internal consistency for the Knowledge and Adherence Practices dimensions and their respective domains in the independent confirmatory subsample. The consistently high estimates obtained using both reliability coefficients provide additional support for the internal consistency of the instrument [34,35]. Test–retest analysis also showed strong temporal stability, with overall intraclass correlation coefficients of 0.946 for Knowledge and 0.940 for Adherence Practices. Domain-level coefficients were similarly strong [36].

However, several reliability coefficients exceeded 0.95, prompting further examination of item redundancy. In the confirmatory subsample, within-domain inter-item correlations were generally high, with mean correlations ranging from approximately 0.78 to 0.92 across the eight domains. The strongest clustering was observed in the Adherence Practices fluid-management domain (mean inter-item correlation approximately 0.92; maximum approximately 0.97), and several very high pairwise correlations were also observed in the Adherence Practices dialysis-treatment and Knowledge dialysis-treatment domains. These findings indicate that some items assess closely related aspects of the same clinical behavior and that partial redundancy cannot be excluded. Nevertheless, the 57-item structure was retained in the present study because the items were developed to preserve coverage of distinct clinically relevant behaviors within dialysis treatment, medication use, fluid management, and nutrition, and because post hoc deletion based on the same development and validation dataset would effectively create a shortened version that has not undergone independent psychometric evaluation. Future studies should therefore evaluate whether selected highly correlated items can be removed while preserving content validity, domain coverage, structural validity, and reliability in an independent sample.

The three-week interval was selected to reduce the potential influence of immediate recall while limiting the likelihood of substantial changes in treatment-related knowledge/awareness and self-reported adherence practices. Nevertheless, the test–retest analysis included only 30 participants. Although the observed ICCs indicated strong temporal stability, the relatively small retest sample limits the precision and generalizability of these estimates, and replication in a larger sample is warranted. In addition, all study instruments were administered through face-to-face interviews; therefore, reliability across alternative administration modes, such as self-administration, was not evaluated. Future studies should examine whether the psychometric performance of the scale is comparable across different modes of administration.

### 4.4. Score Distributions and Exploratory Comparisons

Participants obtained relatively high knowledge scores, and the knowledge dimension and its domains were negatively skewed. This finding may reflect effective patient education and experience gained through self-managing PD. However, it may also indicate social-desirability bias, limited item difficulty, or potential ceiling effects.

Although nurses received standardized instructions to administer the items neutrally, face-to-face administration by peritoneal dialysis nurses may have increased susceptibility to social-desirability bias, acquiescence, and interviewer-related response effects, including a tendency to provide responses perceived as desirable to healthcare providers. This administration effect may have contributed to the concentration of Knowledge scores toward the upper end and should be examined in future studies using alternative administration modes.

Although the Knowledge Dimension showed negative skewness, the formal floor and ceiling analysis demonstrated that no participants achieved the theoretical minimum score, while the proportion reaching the theoretical maximum was no greater than 2.7%. Thus, the distribution did not indicate a substantial floor or ceiling effect at the overall dimension level [27]. The negative skewness therefore appears to reflect a concentration of scores toward the upper end of the scale rather than accumulation at the absolute maximum. Nevertheless, further validation should examine the instrument’s ability to discriminate among patients with relatively high levels of self-reported treatment knowledge.

Formal floor and ceiling effects were evaluated at the overall dimension level rather than separately for each domain. Domain-level score distributions were therefore characterized using their means, standard deviations, skewness, and kurtosis (Table 6). The marked negative skewness observed in several Knowledge domains indicates upper-end score concentration and should be examined further using domain-specific floor and ceiling analyses in future independent validation studies.

Adherence Practices scores showed greater variability than knowledge scores. This supports evaluating the two dimensions separately because understanding the treatment regimen does not necessarily indicate consistent implementation of the recommended treatment practices. An integrative review of self-efficacy in home dialysis similarly emphasized that patients’ confidence develops over time and is influenced by skills, continuing knowledge development, professional support, family involvement, and peer support [41]. Studies involving patients receiving hemodialysis have also identified associations between treatment knowledge, adherence, and quality of life [42], as well as between perceived social support and treatment adherence [43]. Although these findings provide relevant context, they should not be assumed to generalize directly to patients receiving home PD.

Exploratory subgroup analyses identified several differences across selected demographic and clinical characteristics. However, because these analyses were not prespecified, involved multiple comparisons, were not adjusted for potential confounding, and measurement invariance was not established, they should be interpreted as hypothesis-generating rather than as evidence of known-groups validity or independent associations. Future studies should evaluate such subgroup differences using prespecified hypotheses, multivariable analyses, appropriate multiplicity control, and established measurement invariance.

### 4.5. Clinical Implications

The Peritoneal Dialysis Treatment Adherence Scale may support a differentiated patient-reported assessment of treatment-related knowledge/awareness and adherence practices among adults receiving peritoneal dialysis. Because the Knowledge and Adherence Practices dimensions are scored separately, the instrument may help healthcare professionals distinguish limited self-reported treatment knowledge or awareness from less consistent self-reported adherence practices.

The domain scores may indicate areas related to dialysis procedures, medication use, fluid management, or nutrition that warrant further discussion or clinical assessment. Lower Knowledge scores may prompt further assessment of the patient’s understanding of treatment requirements, whereas lower Adherence Practices scores may prompt further assessment of barriers to consistently carrying out recommended treatment practices. Patient-level physical and cognitive barriers, together with the availability of support, can influence patients’ ability to undertake PD-related self-management [44]. Psychological factors should also be considered, as anxiety symptoms have been associated with poorer treatment adherence in dialysis populations [45]. Where broader clinical assessment identifies gaps in treatment knowledge or potential barriers related to beliefs or confidence, individualized educational and nursing interventions may be appropriate; Health Belief Model-informed nursing approaches have demonstrated improvements in self-care among patients receiving maintenance hemodialysis [46]. Family involvement should likewise be assessed individually, because although caregiver support may facilitate home treatment, caregiving in PD can itself be associated with substantial burden [47]. Nutritional assessment is also clinically important in PD, as a retrospective study of patients receiving continuous ambulatory peritoneal dialysis identified associations between nutritional status and hospitalization and mortality outcomes [48]. Studies in hemodialysis populations have additionally documented difficulties across different components of treatment adherence, including dialysis attendance, medication use, dietary recommendations, and fluid restriction [49,50,51], although these findings cannot be directly extrapolated to PD. These findings should not be interpreted as definitive evidence of specific educational, behavioral, or psychosocial needs. The instrument may therefore have potential as an adjunctive tool to support individualized clinical assessment and discussion; however, decisions regarding education or supportive interventions should be based on a broader assessment rather than on scale scores alone.

The present cross-sectional validation study does not establish that use of the scale improves treatment adherence, patient education, care planning, or clinical outcomes. Although administration required approximately 15 min in this study, the 57-item length may limit feasibility in time-constrained routine clinical settings. Future studies should evaluate completion burden and acceptability and determine whether a shorter form can retain adequate psychometric performance and coverage of the four adherence domains.

However, the scale should not be used as a stand-alone measure of actual treatment adherence. Scores should be interpreted alongside patient interviews, medication reviews, dialysis records, supply use, cycler or remote-monitoring data, and relevant clinical and biochemical indicators [3,6,7]. Cutoff scores distinguishing adherence from non-adherence have not yet been established, and the instrument’s responsiveness and predictive value require further evaluation before routine clinical implementation.

### 4.6. Strengths and Limitations

The principal strengths of this study include its multicenter design, large sample of 600 adults receiving PD, inclusion of both public and university hospitals, and use of independent subsamples for exploratory and confirmatory factor analyses. The instrument-development process also included expert evaluation, pilot testing, item analysis, assessment of internal convergent evidence and within-dimension discriminant validity, internal consistency analysis, and test–retest reliability evaluation.

Several limitations should be considered. First, the study was conducted in Türkiye, and the applicability of the factor structure and scoring system to other languages, cultures, and healthcare systems remains unknown. The participating centers were purposively selected to achieve institutional and geographical diversity and may therefore not be representative of all PD centers nationally or internationally. Although the overall response rate was high (95.2%), individual-level demographic and clinical data were not available for patients who declined participation or were excluded because of incomplete questionnaires. Consequently, differences between included and non-included patients could not be formally assessed, and selection bias cannot be excluded. The face-to-face self-report administration method may also have increased susceptibility to recall and social desirability bias, particularly for items concerning treatment-related knowledge/awareness and self-reported adherence practices.

Second, the instrument measures self-reported adherence-related treatment knowledge/awareness and self-reported adherence practices rather than directly observed treatment behavior. Because the Knowledge Dimension uses frequency-based response options rather than objectively scored correct/incorrect items, it should not be interpreted as a direct test of factual knowledge. Scores may partly reflect perceived knowledge or habitual treatment-related response tendencies in addition to understanding of treatment requirements. Formal cognitive interviewing was not conducted, and supplementary objectively scored knowledge items were not included in the original study. Future validation studies should therefore use cognitive interviewing to examine how patients interpret the Knowledge items and should compare Knowledge Dimension scores with objectively scored PD-related knowledge measures and objective indicators of treatment adherence.

Third, the scale was not evaluated against an established adherence instrument, objective treatment data, or clinical outcomes. Criterion validity, predictive validity, responsiveness, and clinically meaningful cutoff scores therefore remain undetermined. Because the present study used a cross-sectional design, it cannot establish whether Knowledge and Adherence Practices scores predict subsequent clinical outcomes or whether the instrument is sensitive to changes over time or following an intervention. Consequently, the present findings do not establish that higher scale scores correspond to better real-world treatment adherence. Longitudinal studies are needed to determine whether the scores predict clinically relevant outcomes such as peritonitis, hospitalization, technique failure, treatment discontinuation, biochemical control, and longer-term treatment adherence. The test–retest analysis included only 30 participants, which limits the precision and generalizability of the stability estimates; replication in a larger sample is therefore warranted. Because the characteristics of the test–retest subsample were not formally compared with those of the full sample, its representativeness could not be established.

In addition, all study instruments were administered through face-to-face interviews, and equivalence of reliability across alternative administration modes, including self-administration, was not evaluated.

Several internal consistency coefficients exceeded 0.95, suggesting that some items may contain overlapping content [34]. However, post hoc reduction of the present 57-item instrument using the same development and validation dataset could capitalize on sample-specific characteristics and would not, by itself, constitute a validated short form. The full version evaluated in the present study was therefore retained. Future research should develop a shorter version using prespecified item-reduction criteria and subsequently evaluate its factor structure, reliability, validity, and measurement invariance in an independent sample. Accordingly, a priority for the next phase of research will be the development and independent validation of a shorter version that preserves the conceptual structure and clinically relevant domain coverage of the original scale.

The Knowledge scores were negatively skewed; however, the floor and ceiling assessment did not indicate a substantial floor or ceiling effect at the overall dimension level [27]. Formal measurement invariance was not established across study sites, demographic groups, or PD modalities. The relatively small numbers of participants within individual centers limited the feasibility of stable center-specific multigroup CFA for the 57-item eight-factor model. In addition, because the integrated CFA did not demonstrate uniformly acceptable global model fit, proceeding to increasingly constrained configural, metric, and scalar invariance models was not considered methodologically appropriate. Consequently, subgroup score comparisons in the present study should be interpreted as exploratory rather than as evidence of measurement-equivalent group differences. Future studies should evaluate configural, metric, and scalar invariance in larger independent samples with adequately sized subgroups before formal comparisons across sites, demographic groups, or PD modalities are undertaken.

The five-point Likert-type response format and the negatively skewed distributions observed in several Knowledge domains should be considered when interpreting the CFA findings. The ordinal sensitivity analysis indicated that the standardized factor-loading pattern remained strong, but residual model fit was partly sensitive to the estimation approach. Accordingly, the CFA findings should be regarded as provisional. Further validation using ordinal-data estimators in an independent sample is warranted.

Finally, the integrated eight-factor CFA was evaluated in the confirmatory subsample, which was independent of the subsample used for EFA. To minimize the risk of data-driven overfitting, the integrated model was estimated without introducing additional correlated residuals or post hoc error covariances solely to improve model fit. Although this provided a more conservative evaluation of the proposed measurement structure, the integrated model did not meet all conventional global-fit criteria, and no third independent or external sample was available for replication. The overall eight-factor measurement structure therefore requires further independent validation before its generalizability can be established.

## 5. Conclusions

This study developed the Peritoneal Dialysis Treatment Adherence Scale, a patient-reported instrument comprising separately scored Knowledge and Adherence Practices dimensions across dialysis treatment, medication use, fluid management, and nutrition. The instrument demonstrated promising but provisional structural evidence, internal convergent evidence, evidence of within-dimension discriminant validity, high internal consistency, and strong temporal stability. Knowledge scores reflect self-reported treatment-related knowledge and awareness, whereas Adherence Practices scores reflect the frequency and consistency of self-reported adherence practices related to recommended PD care.

The instrument may support domain-specific assessment and individualized patient education and nursing care by helping identify areas in which patients report lower treatment-related knowledge/awareness or less consistent adherence practices. However, the scores should be interpreted as continuous measures and should not be used to classify patients as adherent or non-adherent or as substitutes for objective adherence indicators. Further independent validation, including external and predictive validation, is warranted before widespread clinical use.

## Figures and Tables

**Table 1:** Sociodemographic and Clinical Characteristics of Participants (*N* = 600).

**Panel A. Categorical Characteristics**			
**Characteristic**	**Category**	***n***	**%**
Sex	Women	402	67.0
	Men	198	33.0
Marital status	Married	461	76.8
	Single	104	17.3
	Divorced	14	2.3
	Other	21	3.5
Age group, years	20–30	38	6.3
	31–40	144	24.0
	41–50	141	23.5
	51–60	150	25.0
	61–70	86	14.3
	≥71	41	6.8
Educational level	Illiterate	43	7.2
	Literate	64	10.7
	Primary education	288	48.0
	High school	164	27.3
	University	41	6.8
Occupation	Homemaker	263	43.8
	Civil servant	39	6.5
	Worker	42	7.0
	Self-employed	60	10.0
	Other	111	18.5
	Not employed	85	14.2
Place of residence	Village	109	18.2
	City	491	81.8
Living arrangement	With spouse	127	21.2
	With spouse and children	384	64.0
	Alone	89	14.8
Perceived economic status	Sufficient	95	15.8
	Moderate	304	50.7
	Insufficient	201	33.5
PD modality	CAPD	425	70.8
	APD	175	29.2
Previous kidney replacement therapy	Kidney transplantation	48	8.0
	Hemodialysis	199	33.2
	None	353	58.8
Received information on diet/fluid restriction	Yes	431	71.8
	No	169	28.2
Additional chronic disease	Yes	380	63.3
	No	220	36.7
Follows recommended diet	Yes	425	70.8
	No	175	29.2
Uses prescribed medications	Yes	585	97.5
	No	15	2.5
Takes medications regularly	Yes	520	86.7
	No	80	13.3
Performs dialysis treatment regularly	Yes	586	97.7
	No	14	2.3
Regularly monitors fluid balance	Yes	525	87.5
	No	75	12.5
**Panel B. Continuous Characteristics**			
**Characteristic**	**Observed Range**	**Mean**	**SD**
Age, years	23–79	49.34	13.43
Duration of kidney disease, years	1–27	8.62	5.57
Duration of PD treatment, years	1–16	4.42	3.42

Note. APD = automated peritoneal dialysis; CAPD = continuous ambulatory peritoneal dialysis; PD = peritoneal dialysis; SD = standard deviation.

**Table 2:** Item Reduction and Exploratory Factor Analysis Results (First Subsample, *N* = 300).

**Panel A. Item Analysis and Items Reduction**						
**Dimension**	**Initial Items**	**Removed Original Item Numbers**		**Retained Items**		**Corrected Item–Total Correlation Range**
**Knowledge**	39	4, 5, 12, 16, 23, 26, and 37		32		0.679–0.951
**Adherence Practices**	25	None		25		0.667–0.962
**Panel B. Exploratory Factor Analysis**						
**Dimension**	**KMO**	**Bartlett χ^2^ (df)**	***p***	**Factors Retained by Parallel Analysis**	**Primary Factor-Loading Range**	**Communality (h^2^) Range**
**Knowledge**	0.935	18,011.53 (496)	<0.001	4	0.694–0.971	0.649–0.958
**Adherence Practices**	0.885	6103.43 (300)	<0.001	4	0.508–0.951	0.263–0.909

Note. Principal axis factoring with direct oblimin rotation was used. Horn’s parallel analysis supported four factors for both the Knowledge and Adherence Practices dimensions, corresponding to dialysis treatment, medication use, fluid management, and nutrition. KMO = Kaiser–Meyer–Olkin measure of sampling adequacy; h^2^ = communality.

**Table 3:** Model-Fit Indices for the Confirmatory Factor Analyses (*n* = 300).

**Panel A. Separate Four-Factor Models**										
**Model**	**χ^2^/df**	**RMR**	**GFI**	**AGFI**	**NFI**	**IFI**	**TLI**	**CFI**	**RMSEA**	**Interpretation**
Knowledge	2.32	0.068	0.908	0.905	0.957	0.958	0.944	0.957	0.073	Acceptable
Adherence Practices	2.21	0.052	0.903	0.902	0.928	0.950	0.913	0.950	0.067	Acceptable
**Panel B. Integrated Eight-Factor Model**										
**Model**						**χ^2^/df**	**CFI**	**TLI**	**RMSEA**	**SRMR**
Integrated eight-factor model (57 items)						4.22	0.818	0.808	0.104	0.067

Note. Panel A presents the originally specified separate four-factor CFA models for the Knowledge and Adherence Practices dimensions. Panel B presents the additional integrated eight-factor CFA in which all 57 items were analyzed simultaneously and correlations among the eight latent factors were freely estimated. The integrated model was estimated without additional correlated residuals. CFI = comparative fit index; TLI = Tucker–Lewis index; RMSEA = root mean square error of approximation; SRMR = standardized root mean square residual.

**Table 4:** Internal Convergent Evidence and Within-Dimension Discriminant Validity Statistics (CFA Sample, *N* = 300).

**Panel A. Knowledge Dimension.**									
**Factor**	**CR**	**AVE**	**MSV**	**ASV**	**MaxR(H)**	**K–Medication**	**K–Fluid**	**K–Nutrition**	**K–Dialysis**
**K–Medication**	0.964	0.818	0.354	0.288	0.978	**0.904**			
**K–Fluid**	0.953	0.803	0.343	0.243	0.965	0.586	**0.896**		
**K–Nutrition**	0.980	0.842	0.425	0.258	0.986	0.409	0.426	**0.918**	
**K–Dialysis**	0.978	0.874	0.425	0.328	0.982	0.595	0.451	0.652	**0.935**
**Panel B. Adherence Practices Dimension**									
**Factor**	**CR**	**AVE**	**MSV**	**ASV**	**MaxR(H)**	**A–Fluid**	**A–Dialysis**	**A–Medication**	**A** **–** **Nutrition**
**A–Fluid**	0.980	0.893	0.266	0.139	0.988	**0.945**			
**A–Dialysis**	0.978	0.779	0.078	0.058	0.987	0.143	**0.883**		
**A–Medication**	0.940	0.839	0.272	0.205	0.955	0.516	0.274	**0.916**	
**A** **–** **Nutrition**	0.965	0.901	0.272	0.160	0.969	0.359	0.280	0.522	**0.949**

Note. Bold diagonal values are the square roots of AVE; off-diagonal values are inter-factor correlations. K = Knowledge; A = Adherence Practices; ASV = average shared variance; AVE = average variance extracted; CR = composite reliability; MaxR(H) = maximum reliability; MSV = maximum shared variance.

**Table 5:** Internal Consistency and Three-Week Test–Retest Reliability.

Dimension		Domain		Cronbach’s α (95% CI)		McDonald’s ω (95% CI)	
Knowledge		Overall		0.950 (0.940–0.956)		0.984 (0.981–0.987)	
		Dialysis treatment		0.980 (0.972–0.984)		0.980 (0.973–0.985)	
		Medication use		0.954 (0.941–0.964)		0.956 (0.943–0.965)	
		Fluid management		0.920 (0.894–0.939)		0.923 (0.900–0.942)	
		Nutrition		0.966 (0.954–0.974)		0.968 (0.958–0.975)	
Adherence Practices		Overall		0.952 (0.941–0.960)		0.986 (0.983–0.989)	
		Dialysis treatment		0.979 (0.974–0.982)		0.979 (0.975–0.983)	
		Medication use		0.938 (0.916–0.954)		0.939 (0.919–0.955)	
		Fluid management		0.981 (0.976–0.985)		0.982 (0.977–0.986)	
		Nutrition		0.964 (0.948–0.976)		0.965 (0.949–0.977)	
Panel B. Three-Week Test–Retest Reliability (*n* = 30)							
Dimension	Domain	First M ± SD	Second M ± SD	ICC	95% CI	F	*p*
Knowledge	Dialysis Treatment	4.30 ± 0.33	4.32 ± 0.39	0.857	0.699–0.932	6.973	<0.001
	Medication use	4.45 ± 0.20	4.47 ± 0.29	0.931	0.854–0.967	14.394	<0.001
	Fluid management	4.30 ± 0.22	4.36 ± 0.28	0.909	0.808–0.957	10.952	<0.001
	Nutrition	4.36 ± 0.28	4.38 ± 0.40	0.962	0.920–0.982	26.101	<0.001
	Overall	4.35 ± 0.16	4.38 ± 0.20	0.946	0.886–0.974	18.426	<0.001
Adherence Practices	Dialysis Treatment	4.32 ± 0.54	4.35 ± 0.75	0.942	0.878–0.972	17.260	<0.001
	Medication use	4.15 ± 0.27	4.12 ± 0.48	0.845	0.675–0.926	6.457	<0.001
	Fluid management	4.36 ± 0.35	4.38 ± 0.51	0.936	0.865–0.970	15.619	<0.001
	Nutrition	3.99 ± 0.32	3.93 ± 0.47	0.841	0.667–0.924	6.285	<0.001
	Overall	4.21 ± 0.22	4.19 ± 0.33	0.940	0.875–0.972	16.751	<0.001

Note. α = Cronbach’s alpha; ω = McDonald’s omega; CI = confidence interval. Ninety-five percent confidence intervals were obtained using participant-level nonparametric bootstrap resampling (1000 resamples).

**Table 6:** Dimension and Domain Score Distributions (*N* = 600).

Dimension	Score	Theoretical Range	Mean ± SD	Skewness	Kurtosis
Knowledge	Overall	32–160	128.70 ± 31.77	−0.954	−0.133
	Dialysis treatment	12–60	50.99 ± 17.71	−1.540	0.721
	Medication use	6–30	22.62 ± 9.21	−1.091	−0.502
	Fluid management	5–25	19.70 ± 6.51	−1.391	0.675
	Nutrition	9–45	35.39 ± 11.83	−1.548	0.947
Adherence Practices	Overall	25–125	77.82 ± 27.15	−0.456	−0.540
	Dialysis treatment	13–65	38.42 ± 20.47	0.206	−1.651
	Medication use	3–15	11.41 ± 3.83	−1.017	0.078
	Fluid management	6–30	18.05 ± 8.18	−0.252	−1.054
	Nutrition	3–15	9.93 ± 4.57	−0.506	−1.267

Note. Higher scores indicate greater self-reported treatment-related knowledge/awareness or more consistent self-reported adherence practices. SD = standard deviation. Floor/ceiling effects for the overall dimension scores were 0.0%/≤2.7% for Knowledge and 0.0%/0.0% for Adherence Practices.

## Data Availability

The de-identified data supporting the findings of this study are available from the corresponding author upon reasonable request, subject to institutional approval and applicable ethical and data-protection requirements. The data are not publicly available because they contain information collected from human participants and are subject to privacy and ethical restrictions.

## Supplementary Materials

The following supporting information can be downloaded at: https://www.mdpi.com/article/10.3390/healthcare14193338/s1, Table S1: Item-Level Analysis of the Knowledge Dimension (EFA Sample, N = 300); Table S2: Item-Level Analysis of the Adherence Practices Dimension (EFA Sample, N = 300); Table S3: Knowledge Dimension and Domain Scores According to Participant Characteristics (N = 600); Table S4: Adherence Practices Dimension and Domain Scores According to Participant Characteristics (N = 600); Table S5: Final English Questionnaire and Administration/Scoring Guide; Table S6: Pattern Matrices and Communalities from the Exploratory Factor Analysis (Exploratory Subsample, N = 300); Table S7: SStandardized Factor Loadings and Latent-Factor Correlations from the Integrated Eight-Factor Confirmatory Factor Analysis (n = 300); Figure S1: Development and Psychometric Evaluation Process of the Peritoneal Dialysis Treatment Adherence Scale; Figure S2: Standardized Four-Factor CFA Model for the Knowledge Dimension; Figure S3: Standardized Four-Factor CFA Model for the Adherence Practices Dimension.
