# Supplementary material for: Development and Psychometric Evaluation of the Peritoneal Dialysis Treatment Adherence Scale: A Multicenter Methodological Study
Source: Healthcare (Basel). 2026 Oct 7;14(19):3338. doi: 10.3390/healthcare14193338 (PMC13665426; doi:10.3390/healthcare14193338)
Supplement: Supplementary file 1 [file healthcare-14-03338-s001.zip › healthcare-4569768-supplementary.pdf]

SUPPLEMENTARY MATERIALS

Development and Psychometric Evaluation of the Peritoneal Dialysis Treatment Adherence Scale: A Multicenter Methodological Study

Zehra Aydın and Tuğba Pehlivan Sarıbudak

Tables S1–S7

CONTENTS

Table S1 Knowledge dimension: item-level analysis

Table S2 Adherence Practices dimension: item-level analysis

Table S3 Knowledge dimension: participant group comparisons

Table S4 Adherence Practices dimension: participant group comparisons

Table S5 Final questionnaire and user guide

Table S6 Exploratory factor-analysis pattern matrices and communalities

Table S7 Integrated eight-factor CFA loadings and factor correlations

Figure S1. Development and Psychometric Evaluation Process of the Peritoneal Dialysis Treatment Adherence Scale
Figure S2. Standardized Four-Factor CFA Model for the Knowledge Dimension
Figure S3. Standardized Four-Factor CFA Model for the Adherence Practices Dimension

Supplementary Table S1. Item-Level Analysis of the Knowledge Dimension (EFA Sample, N = 300)

Domain	Original item no.	Corrected item–total correlation	Cronbach's α if item deleted
**K–Dialysis**	1	0.847	0.979
K–Dialysis	2	0.826	0.978
K–Dialysis	3	0.920	0.977
K–Dialysis	6	0.849	0.979
K–Dialysis	7	0.932	0.974
K–Dialysis	8	0.937	0.977
K–Dialysis	9	0.878	0.978
K–Dialysis	10	0.897	0.976
K–Dialysis	11	0.864	0.978
K–Dialysis	13	0.887	0.975
K–Dialysis	14	0.850	0.979
K–Dialysis	15	0.951	0.976
**K–Medication**	17	0.853	0.946
K–Medication	18	0.792	0.953
K–Medication	19	0.932	0.937
K–Medication	20	0.823	0.950
K–Medication	21	0.915	0.939
K–Medication	22	0.838	0.948
**K–Fluid**	24	0.782	0.905
K–Fluid	25	0.821	0.897
K–Fluid	27	0.860	0.889
K–Fluid	28	0.841	0.893
K–Fluid	29	0.679	0.926
**K–Nutrition**	30	0.842	0.962
K–Nutrition	31	0.871	0.961
K–Nutrition	32	0.876	0.961
K–Nutrition	33	0.780	0.965
K–Nutrition	34	0.897	0.960
K–Nutrition	35	0.778	0.966
K–Nutrition	36	0.828	0.963
K–Nutrition	38	0.919	0.959
K–Nutrition	39	0.939	0.958

Note. *K = Knowledge; K–Dialysis = Knowledge–Dialysis Treatment; K–Medication = Knowledge–Medication Use; K–Fluid = Knowledge–Fluid Management; K–Nutrition = Knowledge–Nutrition.*

Supplementary Table S2. Item-Level Analysis of the Adherence Practices Dimension (EFA Sample, N = 300)

Domain	Item no.	Corrected item–total correlation	Cronbach's α if item deleted
**A–Dialysis**	1	0.667	0.980
**A–Dialysis**	2	0.954	0.974
**A–Dialysis**	3	0.919	0.975
**A–Dialysis**	4	0.895	0.975
**A–Dialysis**	5	0.928	0.974
**A–Dialysis**	6	0.902	0.975
**A–Dialysis**	7	0.884	0.975
**A–Dialysis**	8	0.900	0.975
**A–Dialysis**	9	0.701	0.979
**A–Dialysis**	10	0.905	0.975
**A–Dialysis**	11	0.947	0.974
**A–Dialysis**	12	0.776	0.978
**A–Dialysis**	13	0.894	0.975
**A–Medication**	1	0.784	0.927
**A–Medication**	2	0.849	0.875
**A–Medication**	3	0.881	0.849
**A–Fluid**	1	0.905	0.978
**A–Fluid**	2	0.946	0.974
**A–Fluid**	3	0.962	0.972
**A–Fluid**	4	0.946	0.974
**A–Fluid**	5	0.919	0.976
**A–Fluid**	6	0.908	0.978
**A–Nutrition**	1	0.922	0.935
**A–Nutrition**	2	0.890	0.959
**A–Nutrition**	3	0.931	0.928

Note. A = Adherence Practices; A–Dialysis = Adherence Practices–Dialysis Treatment; A–Medication = Adherence Practices–Medication Use; A–Fluid = Adherence Practices–Fluid Management; A–Nutrition = Adherence Practices–Nutrition.

Supplementary Table S3. Knowledge Dimension and Domain Scores According to Participant Characteristics (N = 600)

Characteristic/category	n	Overall knowledge, M ± SD	K–Dialysis M ± SD	K–Medication, M ± SD	K–Fluid, M ± SD	K–Nutrition, M ± SD
**Sex**						
Women	402	129.61 ± 30.91	51.42 ± 17.31	22.98 ± 8.93	19.89 ± 6.26	35.31 ± 11.87
Men	198	126.85 ± 33.43	50.10 ± 18.51	21.87 ± 9.74	19.32 ± 7.00	35.55 ± 11.77
Test statistic		t = 1.002	t = 0.858	t = 1.353	t = 0.961	t = −0.233
p value		0.318	0.391	0.177	0.337	0.816
Effect size, Cohen's d		***0.087***	***0.074***	***0.117***	***0.083***	***-0.020***
**Marital status**						
Married	461	128.47 ± 32.73	50.47 ± 18.57	22.73 ± 9.20	19.81 ± 6.54	35.45 ± 11.96
Single	104	130.57 ± 27.14	53.87 ± 13.74	22.53 ± 9.18	18.96 ± 6.32	35.20 ± 11.46
Divorced	14	142.00 ± 13.15	55.71 ± 5.16	26.42 ± 6.33	21.92 ± 5.21	37.92 ± 9.08
Other	21	115.76 ± 36.77	44.95 ± 19.16	18.00 ± 9.95	19.61 ± 7.45	33.19 ± 12.72
Test statistic		F = 2.120	F = 2.210	F = 2.603	F = 1.037	F = 0.469
p value		0.097	0.086	0.051	0.375	0.704
Effect size, partial ηp^2		***0.011***	***0.011***	***0.013***	***0.005***	***0.002***
**Age group, years**						
20–30	38	126.63 ± 30.02	51.97 ± 16.33	21.73 ± 9.30	17.92 ± 7.48	35.00 ± 11.02
31–40	144	131.90 ± 28.12	52.92 ± 15.78	22.79 ± 9.34	19.83 ± 5.96	36.36 ± 11.27
41–50	141	125.92 ± 32.70	50.36 ± 18.82	22.23 ± 9.41	19.02 ± 7.05	34.29 ± 12.80
51–60	150	127.42 ± 33.89	49.04 ± 18.92	22.49 ± 9.20	20.58 ± 6.33	35.30 ± 11.91
61–70	86	129.15 ± 34.01	51.03 ± 17.72	22.81 ± 9.35	20.33 ± 6.19	34.96 ± 12.58
≥71	41	132.70 ± 29.65	52.46 ± 16.96	24.21 ± 8.00	18.83 ± 6.19	37.29 ± 8.89
Test statistic		F = 0.722	F = 0.819	F = 0.388	F = 1.783	F = 0.676
p value		0.607	0.537	0.857	0.114	0.642
Effect size, partial ηp^2		***0.006***	***0.007***	***0.003***	***0.015***	***0.006***
**Educational level**						
Illiterate (1)	43	109.30 ± 34.10	41.23 ± 21.74	19.65 ± 10.37	17.23 ± 7.44	31.18 ± 13.81
Literate (2)	64	140.20 ± 22.38	56.29 ± 10.60	25.93 ± 5.98	20.43 ± 5.58	37.53 ± 10.74

Characteristic/category	n	Overall knowledge, M ± SD	K–Dialysis M ± SD	K–Medication, M ± SD	K–Fluid, M ± SD	K–Nutrition, M ± SD
Primary education (3)	288	128.72 ± 32.26	51.45 ± 17.84	22.64 ± 9.21	19.54 ± 6.62	35.07 ± 12.18
High school (4)	164	130.92 ± 30.85	51.68 ± 17.28	22.39 ± 9.38	20.61 ± 6.12	36.23 ± 10.95
University (5)	41	122.12 ± 32.76	46.92 ± 18.82	21.24 ± 10.21	18.63 ± 6.98	35.31 ± 11.41
Test statistic		F = 7.017	F = 5.509	F = 3.501	F = 2.091	F = 2.156
p value		< 0.001	< 0.001	0.008	0.021	0.073
Effect size, partial ηp²		***0.045***	***0.036***	***0.023***	***0.014***	***0.014***
Tukey post hoc		2, 3 > 1	2, 3 > 1	2 > 1	4 > 1	—
**Living arrangement**						
With spouse	127	131.95 ± 30.46	52.65 ± 16.90	22.97 ± 9.01	20.19 ± 6.31	36.12 ± 11.04
With spouse and children	384	128.04 ± 32.40	50.19 ± 18.49	22.66 ± 9.22	19.77 ± 6.41	35.40 ± 11.85
Alone	89	126.93 ± 30.83	52.05 ± 15.15	21.92 ± 9.52	18.69 ± 7.15	34.25 ± 12.83
Test statistic		F = 0.885	F = 1.110	F = 0.354	F = 1.455	F = 0.652
p value		0.413	0.330	0.702	0.234	0.521
Effect size, partial ηp²		***0.003***	***0.004***	***0.001***	***0.005***	***0.002***
**Duration of kidney disease**						
1–5 years (1)	232	125.68 ± 34.23	48.36 ± 20.12	22.13 ± 9.57	20.06 ± 6.37	35.11 ± 11.72
6–10 years (2)	186	131.26 ± 28.89	53.28 ± 14.93	22.72 ± 9.29	19.36 ± 6.67	35.88 ± 11.64
11–15 years (3)	111	131.95 ± 29.07	53.12 ± 15.37	23.19 ± 8.72	20.24 ± 5.93	35.38 ± 12.16
≥16 years (4)	71	126.80 ± 34.15	50.22 ± 18.53	23.01 ± 8.63	18.57 ± 7.30	34.98 ± 12.36
Test statistic		F = 1.577	F = 3.364	F = 0.408	F = 1.374	F = 0.178
p value		0.194	0.018	0.478	0.250	0.912
Effect size, partial ηp²		***0.008***	***0.017***	***0.002***	***0.007***	***0.001***
Tukey post hoc		—	2 > 1	—	—	—
**Duration of PD treatment**						
1–5 years	434	127.03 ± 32.87	50.23 ± 18.43	22.23 ± 9.44	19.57 ± 6.71	34.99 ± 12.00
6–10 years	116	133.11 ± 28.78	52.89 ± 15.67	23.83 ± 8.55	20.12 ± 5.89	36.25 ± 11.57
11–15 years	50	133.02 ± 27.48	53.14 ± 15.46	23.18 ± 8.56	19.88 ± 6.14	36.82 ± 10.91

Characteristic/category	n	Overall knowledge, M ± SD	K–Dialysis M ± SD	K–Medication, M ± SD	K–Fluid, M ± SD	K–Nutrition, M ± SD
Test statistic		F = 2.187	F = 1.439	F = 1.493	F = 0.338	F = 0.921
p value		0.113	0.238	0.226	0.713	0.399
Effect size, partial ηp²		***0.007***	***0.005***	***0.005***	***0.001***	***0.003***
**PD modality**						
CAPD	425	128.73 ± 31.76	51.03 ± 17.70	22.52 ± 9.11	19.69 ± 6.60	35.34 ± 11.72
APD	175	128.62 ± 31.87	50.88 ± 17.78	—	19.73 ± 6.31	35.49 ± 12.12
Test statistic		t = 0.039	t = 0.098	t = 0.170	t = −0.073	t = −0.135
p value		0.969	0.922	0.865	0.941	0.893
Effect size, Cohen's d		***0.004***	***0.009***	***0.015***	***-0.007***	***-0.012***
**Previous kidney replacement therapy**						
Kidney transplantation	48	126.64 ± 31.37	49.12 ± 19.58	22.64 ± 8.99	20.75 ± 4.75	34.12 ± 12.90
Hemodialysis	199	127.71 ± 34.26	50.20 ± 18.74	23.05 ± 9.20	19.73 ± 6.83	34.71 ± 12.85
None	353	129.54 ± 30.39	51.68 ± 16.84	22.37 ± 9.26	19.70 ± 6.51	35.94 ± 11.05
Test statistic		F = 0.321	F = 0.732	F = 0.350	F = 0.724	F = 0.985
p value		0.726	0.481	0.705	0.485	0.374
Effect size, partial ηp²		***0.001***	***0.002***	***0.001***	***0.002***	***0.003***

Note. Independent-samples t tests were used for two-group comparisons and one-way analysis of variance (ANOVA) with Tukey post hoc tests for comparisons involving three or more groups. Effect sizes are reported as Cohen’s d for two-group comparisons and partial eta-squared (ηp²) for ANOVA comparisons. Tukey-adjusted post hoc comparisons were performed following significant ANOVA results. No global multiplicity correction was applied across the full set of exploratory comparisons; therefore, these findings should be interpreted as hypothesis-generating. APD = automated peritoneal dialysis; CAPD = continuous ambulatory peritoneal dialysis; K = Knowledge; K–Dialysis = Knowledge–Dialysis Treatment; K–Medication = Knowledge–Medication Use; K–Fluid = Knowledge–Fluid Management; K–Nutrition = Knowledge–Nutrition; M = mean; PD = peritoneal dialysis; SD = standard deviation. The APD K–Medication descriptive value is not reported because the supplied analysis table was internally inconsistent for this cell; the corresponding test statistic, p value, and effect-size estimate derived from the reported test statistic are retained as reported/calculated.

Supplementary Table S4. Adherence Practices Dimension and Domain Scores According to Participant Characteristics (N = 600)

Characteristic/category	n	Overall Adherence Practices, M ± SD	A–Dialysis, M ± SD	A–Medication, M ± SD	A–Fluid, M ± SD	A–Nutrition, M ± SD
**Sex**						
Women	402	77.57 ± 26.40	38.64 ± 20.47	11.26 ± 3.72	17.59 ± 8.29	10.07 ± 4.53
Men	198	78.31 ± 28.69	37.96 ± 20.51	11.73 ± 4.04	18.98 ± 7.89	9.63 ± 4.65
Test statistic		t = −0.311	t = 0.382	t = −1.416	t = −1.962	t = 1.130
p value		0.756	0.703	0.157	0.051	0.259
**Effect size (Cohen's d)**		**-0.027**	**0.033**	**-0.123**	**-0.170**	**0.098**
**Marital status**						
Married (1)	461	77.64 ± 27.54	38.91 ± 20.59	11.21 ± 3.84	17.66 ± 8.01	9.85 ± 4.53
Single (2)	104	79.51 ± 25.84	38.39 ± 20.69	12.20 ± 3.70	18.99 ± 8.72	9.93 ± 4.71
Divorced (3)	14	78.71 ± 15.71	27.92 ± 14.12	14.14 ± 1.02	24.71 ± 6.46	11.92 ± 3.83
Other (4)	21	72.71 ± 31.45	34.76 ± 19.71	10.04 ± 4.27	17.57 ± 8.31	10.33 ± 5.12
Test statistic		F = 0.393	F = 1.541	F = 5.217	F = 3.982	F = 0.990
p value		0.758	0.203	0.001	0.008	0.397
Tukey post hoc		—	—	3 > 1, 4	3 > 1	—
**Effect size (partial ηp²)**		**0.002**	**0.008**	**0.026**	**0.020**	**0.005**
**Age group, years**						
20–30 (1)	38	67.65 ± 31.54	30.23 ± 19.56	10.78 ± 5.10	17.52 ± 8.89	9.10 ± 5.16
31–40 (2)	144	70.65 ± 29.96	35.43 ± 20.97	10.78 ± 4.48	15.54 ± 8.60	8.90 ± 5.06
41–50 (3)	141	86.62 ± 24.66	43.46 ± 20.15	12.16 ± 2.96	20.24 ± 8.41	10.74 ± 4.21
51–60 (4)	150	75.45 ± 25.36	35.57 ± 19.58	11.32 ± 3.98	18.70 ± 7.49	9.86 ± 4.33
61–70 (5)	86	80.74 ± 24.51	41.98 ± 19.56	11.65 ± 3.20	16.39 ± 6.87	10.70 ± 4.38
≥71 (6)	41	84.65 ± 21.61	42.07 ± 20.71	11.51 ± 2.97	20.92 ± 7.16	10.14 ± 3.92
Test statistic		F = 7.347	F = 5.073	F = 2.162	F = 7.014	F = 3.179
p value		< 0.001	< 0.001	0.057	< 0.001	0.008
Tukey post hoc		3 > 1, 2, 4	3 > 1, 2, 4	—	6 > 2, 5	6, 3 > 2
**Effect size (partial ηp²)**		**0.058**	**0.041**	**0.018**	**0.056**	**0.026**
**Educational level**						
Illiterate (1)	43	89.13 ± 32.39	47.48 ± 20.31	12.46 ± 4.07	19.25 ± 9.82	9.93 ± 4.71
Literate (2)	64	75.76 ± 26.63	36.84 ± 20.59	10.79 ± 3.77	18.39 ± 7.64	9.73 ± 4.71
Primary education (3)	288	77.48 ± 25.50	37.69 ± 20.39	11.63 ± 3.69	18.16 ± 8.00	9.98 ± 4.42
High school (4)	164	77.73 ± 26.64	37.64 ± 19.73	11.75 ± 3.67	18.10 ± 8.24	10.23 ± 4.66
University (5)	41	71.82 ± 32.90	39.58 ± 22.45	8.43 ± 3.96	15.24 ± 7.88	8.56 ± 4.81
Test statistic		F = 2.494	F = 2.409	F = 8.321	F = 1.488	F = 1.151
p value		0.042	0.048	< 0.001	0.204	0.332
Tukey post hoc		1 > 5	1 > 3, 4	1 > 5	—	—
**Effect size (partial ηp²)**		**0.016**	**0.016**	**0.053**	**0.010**	**0.008**
**Living arrangement**						
With spouse (1)	127	80.89 ± 23.86	40.00 ± 20.20	12.12 ± 3.31	17.88 ± 7.26	10.88 ± 4.14
With spouse and children (2)	384	77.08 ± 28.05	38.06 ± 20.73	11.17 ± 3.91	18.23 ± 8.40	9.59 ± 4.59
Alone (3)	89	76.62 ± 27.59	37.68 ± 19.81	11.42 ± 4.08	17.49 ± 8.47	10.02 ± 4.88
Test statistic		F = 1.044	F = 0.491	F = 2.923	F = 0.334	F = 3.892
p value		0.353	0.612	0.055	0.716	0.021
Tukey post hoc		—	—	—	—	1 > 2
**Effect size (partial ηp²)**		**0.003**	**0.002**	**0.010**	**0.001**	**0.013**

Characteristic/category	n	Overall Adherence Practices, M ± SD	A–Dialysis, M ± SD	A–Medication, M ± SD	A–Fluid, M ± SD	A–Nutrition, M ± SD
**Duration of kidney disease**						
1–5 years (1)	232	78.65 ± 29.15	39.82 ± 20.71	11.15 ± 3.97	17.54 ± 8.26	10.12 ± 4.70
6–10 years (2)	186	77.92 ± 25.60	36.58 ± 19.98	12.27 ± 3.56	19.30 ± 7.99	9.75 ± 4.35
11–15 years (3)	111	77.08 ± 25.09	38.60 ± 21.14	11.43 ± 3.63	17.29 ± 8.70	9.74 ± 4.68
≥16 years (4)	71	75.98 ± 27.85	38.33 ± 19.95	9.98 ± 3.90	17.61 ± 7.32	10.04 ± 4.55
Test statistic		F = 0.209	F = 0.866	F = 6.994	F = 2.132	F = 0.300
p value		0.890	0.459	< 0.001	0.095	0.825
Tukey post hoc		—	—	2 > 1, 4	—	—
**Effect size (partial ηp²)**		**0.001**	**0.004**	**0.034**	**0.011**	**0.002**
**Duration of PD treatment**						
1–5 years (1)	434	79.12 ± 27.53	39.83 ± 20.59	11.45 ± 3.72	17.85 ± 8.10	9.98 ± 4.53
6–10 years (2)	116	74.48 ± 25.73	33.93 ± 19.25	11.69 ± 4.07	18.91 ± 8.33	9.93 ± 4.63
11–15 years (3)	50	74.26 ± 26.56	36.56 ± 20.86	10.40 ± 4.09	17.82 ± 8.54	9.48 ± 4.76
Test statistic		F = 1.811	F = 4.057	F = 2.102	F = 0.795	F = 0.272
p value		0.164	0.018	0.123	0.452	0.762
Tukey post hoc		—	1 > 2	—	—	—
**Effect size (partial ηp²)**		**0.006**	**0.013**	**0.007**	**0.003**	**0.001**
**PD modality**						
CAPD	425	77.47 ± 27.62	37.48 ± 20.34	11.57 ± 3.84	18.48 ± 8.23	9.93 ± 4.56
APD	175	78.66 ± 26.04	40.69 ± 20.67	11.02 ± 3.78	17.01 ± 7.97	9.92 ± 4.53
Test statistic		t = −0.487	t = −1.751	t = 1.592	t = 2.006	t = 0.020
p value		0.627	0.080	0.112	0.045	0.984
**Effect size (Cohen's d)**		**-0.044**	**-0.157**	**0.143**	**0.180**	**0.002**
**Previous kidney replacement therapy**						
Kidney transplantation (1)	48	70.02 ± 30.85	37.56 ± 21.73	10.47 ± 4.59	13.64 ± 7.79	8.33 ± 4.86
Hemodialysis (2)	199	82.71 ± 25.94	41.56 ± 20.16	12.08 ± 3.60	18.41 ± 8.06	10.65 ± 4.38
None (3)	353	76.12 ± 26.91	36.76 ± 20.32	11.17 ± 3.80	18.45 ± 8.14	9.73 ± 4.57
Test statistic		F = 5.996	F = 3.570	F = 5.125	F = 7.740	F = 5.852
p value		0.003	0.029	0.006	< 0.001	0.003
Tukey post hoc		2 > 1, 3	2 > 3	2 > 1, 3	2 > 1	2 > 1
**Effect size (partial ηp²)**		**0.020**	**0.012**	**0.017**	**0.025**	**0.019**

Note. Independent-samples t tests were used for two-group comparisons, and one-way analysis of variance (ANOVA) with Tukey post hoc tests was used for comparisons involving three or more groups. Effect sizes are reported as Cohen's d for two-group comparisons and partial eta-squared (ηp²) for ANOVA comparisons. Tukey-adjusted post hoc comparisons were performed following significant ANOVA results. No global multiplicity correction was applied across the full set of exploratory comparisons; therefore, these findings should be interpreted as hypothesis-generating. A = Adherence Practices; A–Dialysis = Adherence Practices–Dialysis Treatment; A–Medication = Adherence Practices–Medication Use; A–Fluid = Adherence Practices–Fluid Management; A–Nutrition = Adherence Practices–Nutrition; APD = automated peritoneal dialysis; CAPD = continuous ambulatory peritoneal dialysis; M = mean; PD = peritoneal dialysis; SD = standard deviation.

Supplementary Table S5. Final English Questionnaire and Administration/Scoring Guide

Peritoneal Dialysis Treatment Adherence Scale

PART I — RESPONDENT QUESTIONNAIRE

INSTRUCTIONS FOR PATIENTS

These statements concern your awareness of peritoneal dialysis treatment requirements and the treatment practices you report following at home. Read each statement and select one option that best describes how often it applies to you. There are no right or wrong answers to this questionnaire. Please answer every statement based on your own experience.

Participant ID (optional): ______________________ Date (optional): ______________________

Choose one answer per statement: 1 = Never · 2 = Rarely · 3 = Sometimes · 4 = Often · 5 = Always

KNOWLEDGE DIMENSION — 32 STATEMENTS

These statements concern self-reported treatment-related recognition, understanding, and awareness. They are not an objectively scored test of factual knowledge.

Dialysis treatment

Knowledge · 12 items

No.	Statement	Never 1	Rarely 2	Sometimes 3	Often 4	Always 5
1	I know that performing dialysis regularly is very important.	☐	☐	☐	☐	☐
2	I know I need to undergo dialysis because my kidneys no longer function.	☐	☐	☐	☐	☐
3	I know that following the dialysis schedule regularly is necessary to stay healthy.	☐	☐	☐	☐	☐
4	I do not believe it is important to perform dialysis at the exact scheduled time.	☐	☐	☐	☐	☐
5	I do not believe regular handwashing is necessary in dialysis.	☐	☐	☐	☐	☐
6	I know that performing catheter dressing in dialysis is important.	☐	☐	☐	☐	☐
7	I believe wearing a mask during dialysis is important.	☐	☐	☐	☐	☐
8	I know that regular hospital check-ups are important.	☐	☐	☐	☐	☐
9	I know I should not run out of dialysis solutions and other materials.	☐	☐	☐	☐	☐
10	I know the importance of using a new mask for each session.	☐	☐	☐	☐	☐
11	I know the importance of using a new cap for each session.	☐	☐	☐	☐	☐
12	I know that I should perform dialysis in an appropriate environment.	☐	☐	☐	☐	☐

Medication use

Knowledge · 6 items

No.	Statement	Never 1	Rarely 2	Sometimes 3	Often 4	Always 5
13	I know I should take the medications prescribed to me because my kidneys do not function.	☐	☐	☐	☐	☐
14	I know I need to take medications regularly to keep my body healthy.	☐	☐	☐	☐	☐
15	I know the health problems that may occur if I do not take my medications.	☐	☐	☐	☐	☐
16	If I do not take my medications, I may be hospitalized.	☐	☐	☐	☐	☐
17	I believe taking my medications is not very important.	☐	☐	☐	☐	☐
18	I know the importance of getting my medications prescribed regularly.	☐	☐	☐	☐	☐

Fluid management

Knowledge · 5 items

No.	Statement	Never 1	Rarely 2	Sometimes 3	Often 4	Always 5
19	I know the importance of monitoring fluid intake.	☐	☐	☐	☐	☐
20	I know that restricting fluid intake is important because my kidneys do not function.	☐	☐	☐	☐	☐
21	I know that excessive fluid intake may cause health issues (e.g., high blood pressure, shortness of breath).	☐	☐	☐	☐	☐
22	If I drink too much fluid, I may be hospitalized.	☐	☐	☐	☐	☐
23	I do not believe limiting fluid intake is very important.	☐	☐	☐	☐	☐

Nutrition

Knowledge · 9 items

No.	Statement	Never 1	Rarely 2	Sometimes 3	Often 4	Always 5
24	I know that paying attention to what I eat is very important.	☐	☐	☐	☐	☐
25	I know that I must follow a proper nutrition/diet plan because my kidneys do not work.	☐	☐	☐	☐	☐
26	I know that I must follow a diet to keep my body healthy.	☐	☐	☐	☐	☐
27	If I eat foods I shouldn’t, I may be hospitalized.	☐	☐	☐	☐	☐
28	I do not believe paying attention to my diet is very important.	☐	☐	☐	☐	☐
29	I know I should consume enough protein.	☐	☐	☐	☐	☐
30	I know the harms of consuming salt (e.g., it raises blood pressure).	☐	☐	☐	☐	☐
31	I know the importance of consuming foods high in fiber.	☐	☐	☐	☐	☐
32	I know the importance of exercising.	☐	☐	☐	☐	☐

ADHERENCE PRACTICES DIMENSION — 25 STATEMENTS

These statements concern the frequency and consistency of self-reported adherence practices related to recommended peritoneal dialysis care.

Dialysis treatment

Adherence Practices · 13 items

No.	Statement	Never 1	Rarely 2	Sometimes 3	Often 4	Always 5
33	I follow my dialysis schedule without skipping any sessions.	☐	☐	☐	☐	☐
34	I complete each dialysis session for the full prescribed duration.	☐	☐	☐	☐	☐
35	I fill my abdomen with the full amount of dialysis solution as recommended.	☐	☐	☐	☐	☐
36	I drain the full amount of dialysis solution from my abdomen each time.	☐	☐	☐	☐	☐
37	I always wash my hands before performing the dialysis procedure.	☐	☐	☐	☐	☐
38	I use antiseptic solution before each dialysis exchange.	☐	☐	☐	☐	☐
39	I perform my catheter dressing at the recommended time intervals.	☐	☐	☐	☐	☐
40	I always wear a mask during dialysis procedures.	☐	☐	☐	☐	☐
41	I regularly attend my scheduled hospital check-ups.	☐	☐	☐	☐	☐
42	I do not cancel my hospital check-up appointments unnecessarily.	☐	☐	☐	☐	☐
43	I ensure that I do not run out of dialysis solutions or necessary materials.	☐	☐	☐	☐	☐
44	I bring my dialysis record log to the hospital when required.	☐	☐	☐	☐	☐
45	I perform my dialysis treatments in a clean and appropriate environment.	☐	☐	☐	☐	☐

Medication use

Adherence Practices · 3 items

No.	Statement	Never 1	Rarely 2	Sometimes 3	Often 4	Always 5
46	I take my medications exactly as prescribed.	☐	☐	☐	☐	☐
47	I never skip or reduce medication doses without approval.	☐	☐	☐	☐	☐
48	I ensure that I always have my prescribed medications available.	☐	☐	☐	☐	☐

Fluid management

Adherence Practices · 6 items

No.	Statement	Never 1	Rarely 2	Sometimes 3	Often 4	Always 5
49	I follow the fluid restriction guidelines given by my healthcare team.	☐	☐	☐	☐	☐
50	I never forgot to record fluid I consume and eliminate.	☐	☐	☐	☐	☐
51	I measure the amount of fluid I consume and eliminate.	☐	☐	☐	☐	☐
52	I regularly record my fluid intake and output.	☐	☐	☐	☐	☐
53	I monitor my body weight consistently.	☐	☐	☐	☐	☐
54	I measure my blood pressure regularly.	☐	☐	☐	☐	☐

Nutrition

Adherence Practices · 3 items

No.	Statement	Never 1	Rarely 2	Sometimes 3	Often 4	Always 5
55	I follow my prescribed diet/nutrition plan.	☐	☐	☐	☐	☐
56	I follow the exercise program recommended to me.	☐	☐	☐	☐	☐
57	I engage in regular physical activity such as walking.	☐	☐	☐	☐	☐

PART II — ADMINISTRATION AND SCORING GUIDE

For researchers and healthcare professionals

ADMINISTRATION NOTE

Part I is the respondent-facing questionnaire. Part II provides administration, scoring, and interpretation guidance and should be retained by the researcher or clinician.

1. Purpose and interpretation

The scale contains two separately scored patient-reported dimensions: Knowledge (32 items) and Adherence Practices (25 items). Both cover dialysis treatment, medication use, fluid management, and nutrition. Knowledge represents self-reported treatment-related recognition, understanding, and awareness—not objectively tested factual knowledge. Adherence Practices reflects the frequency and consistency of self-reported treatment practices and is not a separate measure of motivation, beliefs, confidence, or self-efficacy.

The scale has no combined 57-item score and was not validated as an objective measure of treatment adherence. Scores must not be used alone to classify a patient as adherent or non-adherent.

2. Population and administration

The original methodological study enrolled 600 adults receiving peritoneal dialysis at eight hospitals in Türkiye. In that study, trained peritoneal dialysis nurses administered the items through standardized face-to-face interviews during routine monthly visits. If reading assistance is necessary, read the item and five response options verbatim without suggesting or interpreting an answer. Administration mode may affect responses and should be reported in future studies.

3. Response options and coding

Never	Rarely	Sometimes	Often	Always
1	2	3	4	5

4. Dimensions, item assignments, and score ranges

Dimension	Domain	Final item Nos.	Score range	Reverse-scored Nos.
Knowledge	Dialysis treatment	1–12	12–60	4, 5
Knowledge	Medication use	13–18	6–30	17
Knowledge	Fluid management	19–23	5–25	23
Knowledge	Nutrition	24–32	9–45	28
**Knowledge**	**TOTAL**	**1–32**	**32–160**	**5 items**
Adherence Practices	Dialysis treatment	33–45	13–65	None
Adherence Practices	Medication use	46–48	3–15	None
Adherence Practices	Fluid management	49–54	6–30	None
Adherence Practices	Nutrition	55–57	3–15	None
**Adherence Practices**	**TOTAL**	**33–57**	**25–125**	**None**

Final item numbers 1–57 in Part I are provided for administration and scoring. They differ from the original 64-item pool numbering used in some item-analysis tables. The source item wording and order have been retained.

5. Reverse scoring

Reverse-score the five negatively worded Knowledge statements: items 4, 5, 17, 23, and 28. Use the mapping 1→5, 2→4, 3→3, 4→2, and 5→1 (reverse-coded score = 6 − original response). No Adherence Practices items require reverse scoring. Complete reverse coding before calculating domain and dimension sums.

6. Calculate and record scores

Sum the appropriately coded items within each domain. Sum the four Knowledge domain scores for the Knowledge total and the four Adherence Practices domain scores for the Adherence Practices total. Do not sum Knowledge and Adherence Practices together. For example, Knowledge domain scores of 48 (dialysis), 24 (medication), 20 (fluid), and 36 (nutrition) yield an overall Knowledge score of 128.

Score to record	Possible range	Observed score
Knowledge — Dialysis	12–60	________
Knowledge — Medication	6–30	________
Knowledge — Fluid	5–25	________
Knowledge — Nutrition	9–45	________
**Knowledge TOTAL**	**32–160**	________
Adherence Practices — Dialysis	13–65	________
Adherence Practices — Medication	3–15	________
Adherence Practices — Fluid	6–30	________
Adherence Practices — Nutrition	3–15	________
**Adherence Practices TOTAL**	**25–125**	________

7. Score interpretation and limitations

Higher Knowledge scores indicate greater self-reported recognition, understanding, and awareness of peritoneal dialysis treatment requirements, recommended practices, and potential consequences. Higher Adherence Practices scores indicate greater frequency and consistency of self-reported adherence practices related to recommended peritoneal dialysis care. No validated cutoff scores are available. Scores should be interpreted continuously within their respective dimensions and considered alongside a broader clinical assessment; an individual score does not establish objectively verified adherence.

8. Missing responses

No validated prorating or item-level imputation procedure was established in the original study. Calculate a domain score only when all items in that domain are answered; calculate a dimension total only when all items in that dimension are answered. Report any alternative missing-data procedure explicitly if one is used in subsequent research.

9. Validation context and version control

The final instrument retained 57 of the original 64 items. The original 600-participant sample was randomly divided into independent 300-person exploratory and confirmatory subsamples, and three-week test–retest reliability was evaluated in 30 participants. The separate four-factor Knowledge and Adherence Practices CFA models showed acceptable fit in the primary analyses; however, ordinal sensitivity analysis indicated that residual model fit was partly sensitive to the treatment of the ordinal indicators, particularly for the Knowledge model. The integrated eight-factor CFA did not meet all conventional global-fit criteria. Accordingly, the CFA findings should be regarded as provisional and require further evaluation using ordinal-data estimators in an independent sample. The integrated structure and external criterion-related validity also require further evaluation. For full psychometric results, see the accompanying manuscript.

Guide version: September 2026. Item statements are reproduced from the supplied English scale; sequential item numbers were added for ease of administration and scoring.

Supplementary Table S6. Pattern Matrices and Communalities from the Exploratory Factor Analysis (Exploratory Subsample, N = 300)

Panel A. Knowledge Dimension (32 retained items)

Original item no.	Prespecified domain	Dialysis treatment	Medication use	Fluid management	Nutrition	Communality (h^2)
1	Dialysis treatment	**0.907**	-0.035	0.044	-0.000	0.826
2	Dialysis treatment	**0.836**	0.092	0.014	0.038	0.833
3	Dialysis treatment	**0.971**	0.004	0.056	-0.054	0.932
6	Dialysis treatment	**0.725**	0.076	-0.116	0.320	0.891
7	Dialysis treatment	**0.927**	0.007	-0.037	0.082	0.933
8	Dialysis treatment	**0.957**	0.014	-0.024	0.024	0.937
9	Dialysis treatment	**0.945**	-0.031	0.051	-0.022	0.883
10	Dialysis treatment	**0.935**	0.034	-0.026	0.024	0.913
11	Dialysis treatment	**0.856**	0.061	0.090	-0.015	0.846
13	Dialysis treatment	**0.892**	0.004	0.033	0.044	0.873
14	Dialysis treatment	**0.839**	0.030	0.093	0.011	0.815
15	Dialysis treatment	**0.947**	0.012	-0.030	0.063	0.958
17	Medication use	0.003	**0.856**	-0.033	0.194	0.858
18	Medication use	-0.188	**0.933**	-0.067	0.208	0.814
19	Medication use	0.056	**0.899**	0.122	-0.063	0.943
20	Medication use	0.063	**0.817**	0.160	-0.108	0.814
21	Medication use	0.204	**0.830**	0.082	-0.090	0.907
22	Medication use	0.397	**0.714**	-0.001	-0.092	0.856
24	Fluid management	0.145	0.014	**0.789**	0.099	0.829
25	Fluid management	-0.049	0.042	**0.875**	0.122	0.852
27	Fluid management	-0.003	-0.003	**0.937**	0.038	0.897
28	Fluid management	0.018	-0.034	**0.950**	-0.057	0.852
29	Fluid management	-0.016	0.112	**0.749**	0.007	0.649
30	Nutrition	0.213	-0.098	-0.075	**0.841**	0.857
31	Nutrition	-0.008	0.058	0.066	**0.890**	0.870
32	Nutrition	-0.069	0.176	0.084	**0.884**	0.907
33	Nutrition	0.088	-0.080	0.040	**0.809**	0.724
34	Nutrition	0.022	0.006	0.120	**0.895**	0.917
35	Nutrition	0.256	0.010	-0.017	**0.694**	0.754
36	Nutrition	-0.025	0.016	-0.044	**0.942**	0.845
38	Nutrition	0.008	0.049	0.048	**0.915**	0.914
39	Nutrition	0.034	-0.037	0.019	**0.958**	0.945

Panel B. Adherence Practices Dimension (25 items)

Original item no.	Prespecified domain	Dialysis treatment	Medication use	Fluid management	Nutrition	Communality (h^2)
1	Dialysis treatment	**0.528**	-0.044	-0.151	0.010	0.308
2	Dialysis treatment	**0.951**	-0.034	0.016	-0.025	0.900
3	Dialysis treatment	**0.905**	-0.001	0.049	-0.013	0.816
4	Dialysis treatment	**0.840**	-0.025	0.048	-0.014	0.704
5	Dialysis treatment	**0.898**	0.023	-0.064	0.021	0.819
6	Dialysis treatment	**0.844**	0.025	-0.089	0.055	0.735
7	Dialysis treatment	**0.774**	-0.014	-0.106	0.035	0.620
8	Dialysis treatment	**0.859**	0.007	-0.019	0.014	0.742
9	Dialysis treatment	**0.508**	0.061	0.068	-0.009	0.263
10	Dialysis treatment	**0.834**	-0.024	-0.005	0.015	0.698
11	Dialysis treatment	**0.866**	-0.033	0.109	-0.017	0.755
12	Dialysis treatment	**0.652**	0.061	0.032	0.019	0.432
13	Dialysis treatment	**0.864**	0.043	0.025	-0.036	0.743
14	Medication use	0.101	**0.639**	0.024	-0.065	0.425
15	Medication use	-0.019	**0.851**	0.040	0.022	0.721
16	Medication use	-0.035	**0.806**	-0.056	0.011	0.658
17	Fluid management	0.009	0.033	**0.692**	-0.012	0.474
18	Fluid management	0.016	0.072	**0.785**	-0.010	0.611
19	Fluid management	0.013	-0.008	**0.934**	-0.069	0.847
20	Fluid management	-0.029	0.004	**0.768**	0.054	0.615
21	Fluid management	-0.009	-0.128	**0.621**	0.019	0.418
22	Fluid management	-0.032	-0.050	**0.626**	0.277	0.560
23	Nutrition	0.043	0.079	0.028	**0.806**	0.667
24	Nutrition	-0.024	-0.004	-0.040	**0.821**	0.657
25	Nutrition	0.006	-0.043	0.021	**0.944**	0.909

Note. Principal axis factoring with direct oblimin rotation was used. The four columns represent the prespecified domains. Primary factor loadings are shown in bold. Secondary loadings ≥ 0.30 were examined as potential cross-loadings; a cross-loading was considered problematic when the difference between the primary and secondary loading was < 0.20. Two Knowledge items showed secondary loadings ≥ 0.30 (original item 6: 0.320 on Nutrition; original item 22: 0.397 on Dialysis treatment), but neither met the criterion for problematic cross-loading. No Adherence Practices item showed a problematic cross-loading. h^2 = communality.

Supplementary Table S7. Standardized Factor Loadings and Latent-Factor Correlations from the Integrated Eight-Factor Confirmatory Factor Analysis (n = 300)

Panel A. Standardized factor loadings

Factor	Item within factor	Domain	Standardized loading
K- Dialysis	1	Knowledge - Dialysis treatment	0.888
K- Dialysis	2	Knowledge - Dialysis treatment	0.894
K- Dialysis	3	Knowledge - Dialysis treatment	0.938
K- Dialysis	4	Knowledge - Dialysis treatment	0.856
K- Dialysis	5	Knowledge - Dialysis treatment	0.965
K- Dialysis	6	Knowledge - Dialysis treatment	0.964
K- Dialysis	7	Knowledge - Dialysis treatment	0.929
K- Dialysis	8	Knowledge - Dialysis treatment	0.956
K- Dialysis	9	Knowledge - Dialysis treatment	0.937
K- Dialysis	10	Knowledge - Dialysis treatment	0.930
K- Dialysis	11	Knowledge - Dialysis treatment	0.899
K- Dialysis	12	Knowledge - Dialysis treatment	0.986
K- Medication	1	Knowledge - Medication use	0.868
K- Medication	2	Knowledge - Medication use	0.872
K- Medication	3	Knowledge - Medication use	0.972
K- Medication	4	Knowledge - Medication use	0.929
K- Medication	5	Knowledge - Medication use	0.981
K- Medication	6	Knowledge - Medication use	0.944
K-Fluid	1	Knowledge - Fluid management	0.806
K-Fluid	2	Knowledge - Fluid management	0.905
K-Fluid	3	Knowledge - Fluid management	0.978
K-Fluid	4	Knowledge - Fluid management	0.926
K-Fluid	5	Knowledge - Fluid management	0.807
K-Nutrition	1	Knowledge - Nutrition	0.902
K-Nutrition	2	Knowledge - Nutrition	0.877
K-Nutrition	3	Knowledge - Nutrition	0.850
K-Nutrition	4	Knowledge - Nutrition	0.806
K-Nutrition	5	Knowledge - Nutrition	0.862
K-Nutrition	6	Knowledge - Nutrition	0.912
K-Nutrition	7	Knowledge - Nutrition	0.885
K-Nutrition	8	Knowledge - Nutrition	0.911
K-Nutrition	9	Knowledge - Nutrition	0.945
A- Dialysis	1	Adherence Practices - Dialysis treatment	0.817

Factor	Item within factor	Domain	Standardized loading
A- Dialysis	2	Adherence Practices - Dialysis treatment	0.979
A- Dialysis	3	Adherence Practices - Dialysis treatment	0.976
A- Dialysis	4	Adherence Practices - Dialysis treatment	0.954
A- Dialysis	5	Adherence Practices - Dialysis treatment	0.976
A- Dialysis	6	Adherence Practices - Dialysis treatment	0.955
A- Dialysis	7	Adherence Practices - Dialysis treatment	0.940
A- Dialysis	8	Adherence Practices - Dialysis treatment	0.946
A- Dialysis	9	Adherence Practices - Dialysis treatment	0.841
A- Dialysis	10	Adherence Practices - Dialysis treatment	0.959
A- Dialysis	11	Adherence Practices - Dialysis treatment	0.975
A- Dialysis	12	Adherence Practices - Dialysis treatment	0.874
A- Dialysis	13	Adherence Practices - Dialysis treatment	0.949
A-Medication	1	Adherence Practices - Medication use	0.879
A-Medication	2	Adherence Practices - Medication use	0.949
A-Medication	3	Adherence Practices - Medication use	0.970
A-Fluid	1	Adherence Practices - Fluid management	0.947
A-Fluid	2	Adherence Practices - Fluid management	0.972
A-Fluid	3	Adherence Practices - Fluid management	0.989
A-Fluid	4	Adherence Practices - Fluid management	0.967
A-Fluid	5	Adherence Practices - Fluid management	0.946
A-Fluid	6	Adherence Practices - Fluid management	0.935
A-Nutrition	1	Adherence Practices - Nutrition	0.973
A-Nutrition	2	Adherence Practices - Nutrition	0.933
A-Nutrition	3	Adherence Practices - Nutrition	0.962

Panel B. Latent-factor correlation matrix

Factor	K-Dialysis	K-Medication	K-Fluid	K-Nutrition	A-Dialysis	A-Medication	A-Fluid	A- Nutrition
**K-Dialysis**	1.000	0.517	0.161	0.585	0.089	0.087	0.033	0.084
**K-Medication**	0.517	1.000	0.463	0.221	-0.010	-0.016	-0.020	0.098
**K-Fluid**	0.161	0.463	1.000	0.058	-0.096	-0.019	-0.093	-0.039
**K-Nutrition**	0.585	0.221	0.058	1.000	0.024	0.091	0.002	0.016
**A-Dialysis**	0.089	-0.010	-0.096	0.024	1.000	0.304	0.162	0.332
**A-Medication**	0.087	-0.016	-0.019	0.091	0.304	1.000	0.557	0.502
**A-Fluid**	0.033	-0.020	-0.093	0.002	0.162	0.557	1.000	0.341
**A- Nutrition**	0.084	0.098	-0.039	0.016	0.332	0.502	0.341	1.000

Note. The integrated CFA simultaneously included all 57 retained items across the four Knowledge and four Adherence Practices domains. Each item was specified to load on its prespecified factor, and correlations among the eight latent factors were freely estimated. Knowledge and Adherence Practices remain separately scored dimensions; no combined total score is calculated. K = Knowledge; A = Adherence Practices; K–Dialysis = Knowledge–Dialysis Treatment; K–Medication = Knowledge–Medication Use; K–Fluid = Knowledge–Fluid Management; K–Nutrition = Knowledge–Nutrition; A–Dialysis = Adherence Practices–Dialysis Treatment; A–Medication = Adherence Practices–Medication Use; A–Fluid = Adherence Practices–Fluid Management; A–Nutrition = Adherence Practices–Nutrition.

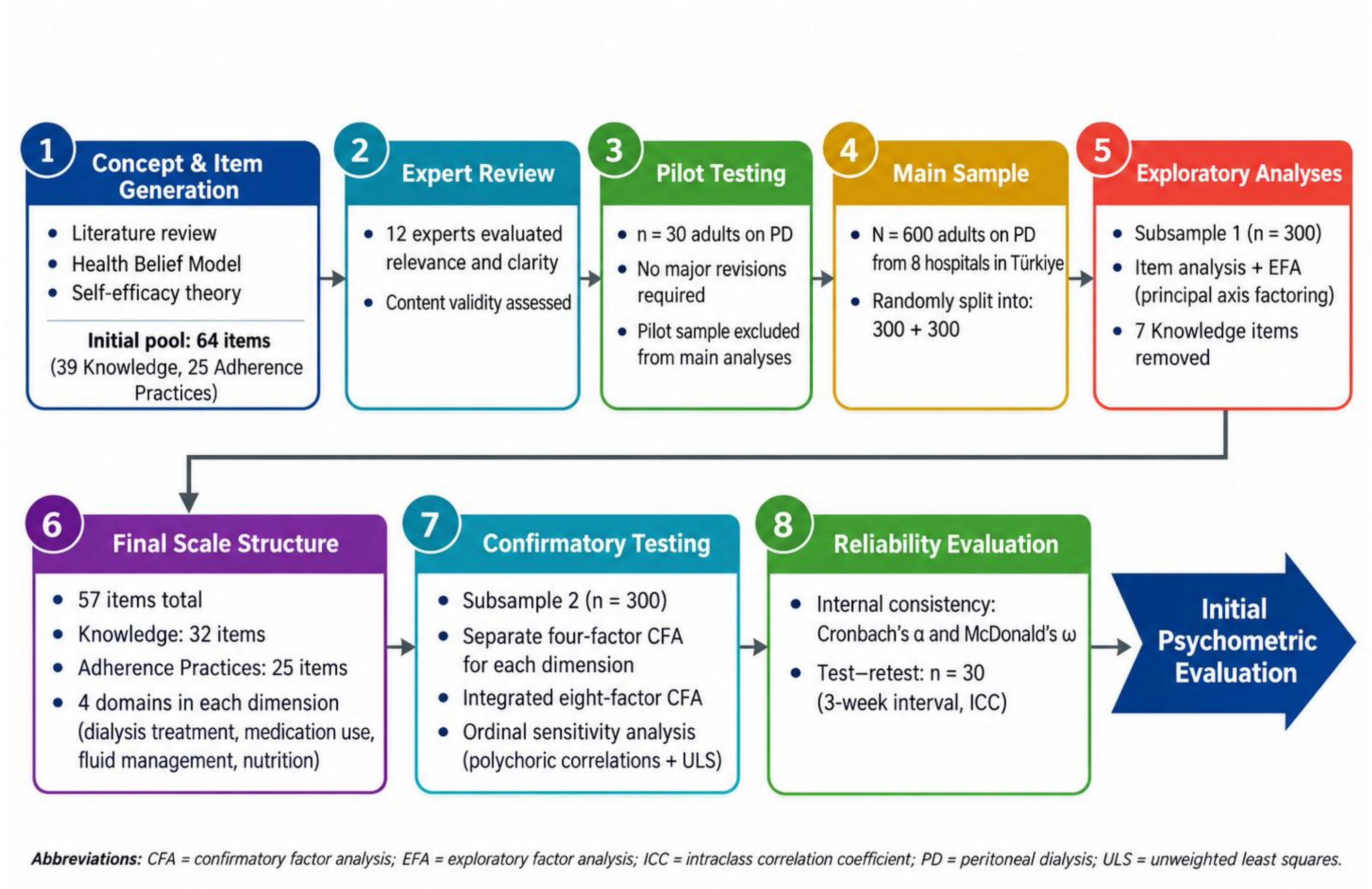

Supplementary Figure S1. Development and Psychometric Evaluation Process of the Peritoneal Dialysis Treatment Adherence Scale

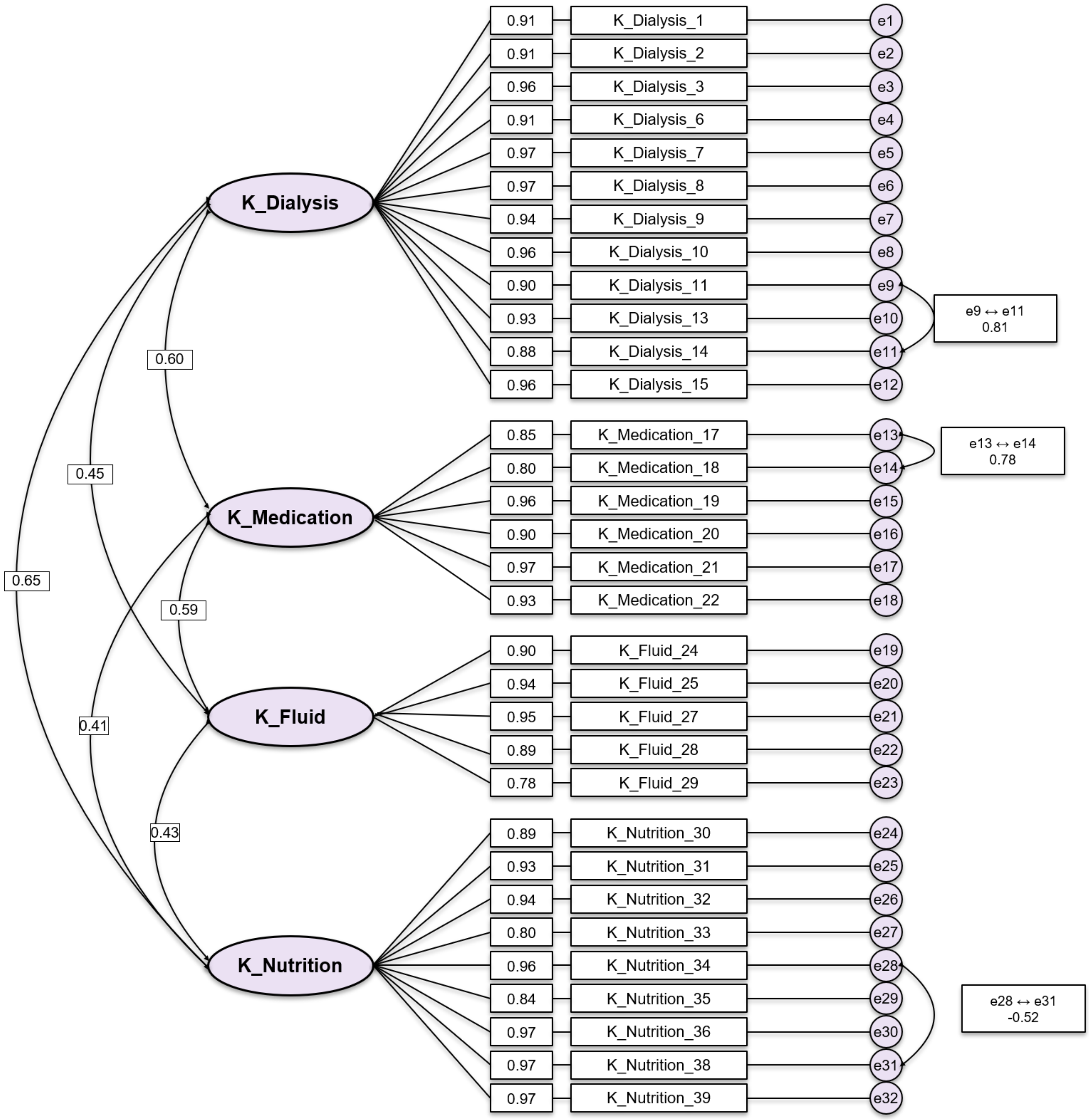

Supplementary Figure S2. Standardized Four-Factor CFA Model for the Knowledge Dimension

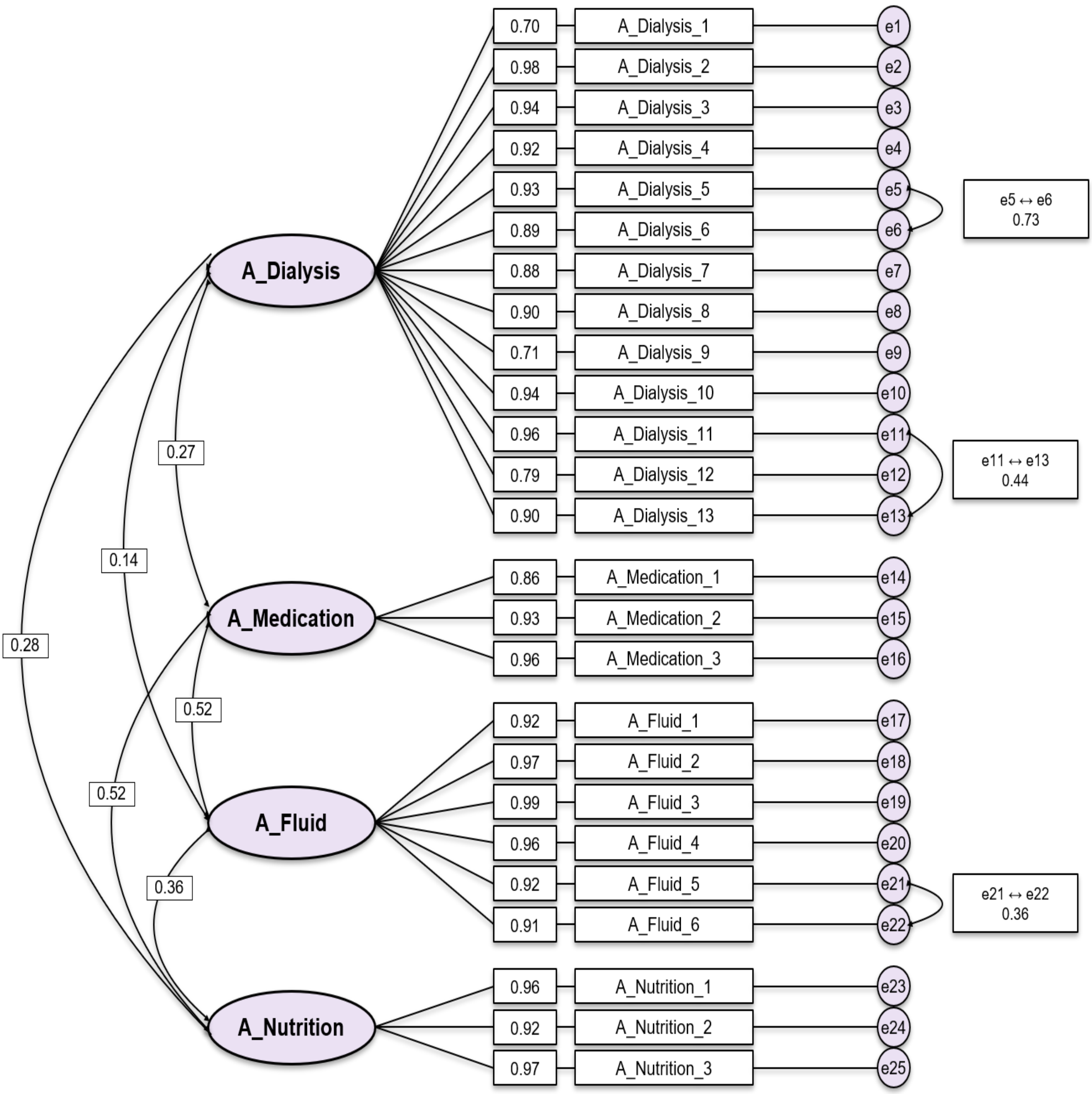

Supplementary Figure S3. Standardized Four-Factor CFA Model for the Adherence Practices Dimension
